# Profiling *Mycobacterium tuberculosis* transmission and the resulting disease burden in the five highest tuberculosis burden countries

**DOI:** 10.1186/s12916-019-1452-0

**Published:** 2019-11-22

**Authors:** Romain Ragonnet, James M. Trauer, Nicholas Geard, Nick Scott, Emma S. McBryde

**Affiliations:** 1 Faculty of Medicine, Dentistry and Health Sciences, The University of Melbourne, Parkville, VIC Australia; 2 School of Public Health and Preventive Medicine, Monash University, 553 St Kilda Road, Melbourne, VIC 3004 Australia; 3 Burnet Institute, 85 Commercial Road, Melbourne, VIC Australia; 4 Victorian Tuberculosis Program at the Peter Doherty Institute for Infection and Immunity, Melbourne, VIC Australia; 5 School of Computing and Information Systems, Melbourne School of Engineering, The University of Melbourne, Melbourne, VIC Australia; 6 The Peter Doherty Institute for Infection and Immunity, The Royal Melbourne Hospital and The University of Melbourne, Melbourne, VIC Australia; 7 Australian Institute of Tropical Health and Medicine, James Cook University, Townsville, Queensland Australia

**Keywords:** Tuberculosis, Transmission profile, Infectious disease, Social mixing

## Abstract

**Background:**

Tuberculosis (TB) control efforts are hampered by an imperfect understanding of TB epidemiology. The true age distribution of disease is unknown because a large proportion of individuals with active TB remain undetected. Understanding of transmission is limited by the asymptomatic nature of latent infection and the pathogen’s capacity for late reactivation. A better understanding of TB epidemiology is critically needed to ensure effective use of existing and future control tools.

**Methods:**

We use an agent-based model to simulate TB epidemiology in the five highest TB burden countries—India, Indonesia, China, the Philippines and Pakistan—providing unique insights into patterns of transmission and disease. Our model replicates demographically realistic populations, explicitly capturing social contacts between individuals based on local estimates of age-specific contact in household, school and workplace settings. Time-varying programmatic parameters are incorporated to account for the local history of TB control.

**Results:**

We estimate that the 15–19-year-old age group is involved in more than 20% of transmission events in India, Indonesia, the Philippines and Pakistan, despite representing only 5% of the local TB incidence. According to our model, childhood TB represents around one fifth of the incident TB cases in these four countries. In China, three quarters of incident TB were estimated to occur in the ≥ 45-year-old population. The calibrated per-contact transmission risk was found to be similar in each of the five countries despite their very different TB burdens.

**Conclusions:**

Adolescents and young adults are a major driver of TB in high-incidence settings. Relying only on the observed distribution of disease to understand the age profile of transmission is potentially misleading.

## Background

Tuberculosis (TB) is now the leading cause of death worldwide from a single infectious agent [1]. While effective prevention and treatment tools have been available for many decades, their impact on the global epidemic has been limited by challenges that TB control programs still face today. Among them, the difficulties in identifying diseased individuals and providing them with adequate care may be the most critical, with only 61% of cases receiving effective treatment [1]. Even more alarming is that the global case detection rate could be as low as 35% in children [2]. As well as ensuring that control policies are as effective as possible, comprehensive knowledge of the epidemic age-profile is essential for estimating burden of disease and predicting the course of the epidemic.

TB epidemiology is also clouded by the propensity of *Mycobacterium tuberculosis* (*M.tb*) to enter a latent infection state within its host (latent TB infection, LTBI), in which it may persist for many years before reactivating [3]. Source tracing is therefore difficult due to the unknown time lag between infection and activation, making transmission events even more challenging to infer than disease burden. While modelling estimates of the global prevalence of LTBI were recently published [4], better understanding pathogen transmission in the population and the resulting infection burden would enable better targeting of high-risk groups.

The slow dynamics of TB limit the feasibility of field investigations that could build epidemic knowledge and mean that historical trends for many decades into the past may have significance for the modern epidemic. For these reasons, mathematical modelling provides a valuable tool to investigate hidden features of the disease [5]. In particular, agent-based models, which explicitly simulate each individual in a population, together with their demographic characteristics, social contacts and infection history, capture important heterogeneities present in real-world populations [6, 7]. This faculty is critical when modelling TB, as *M.tb* transmission is subject to important heterogeneity in characteristics of the infectious host, susceptible host and environment [8]. Meanwhile, the recent availability of contact survey data has dramatically improved our understanding of social mixing [9–11]. In particular, estimates of age-specific contact frequency and intensity in different contexts/locations are now publicly available and provide empiric evidence of preferential mixing patterns, such as age assortativity. Agent-based models can capture specific patterns of social mixing with a high degree of fidelity. For example, they can account for contact saturation in households and other settings such as schools and workplaces where repeated contact is frequent [12]. Incorporating such contacts into a model can enable more accurate estimates of setting-specific contribution to transmission [13].

In this study, we combine data on social mixing and population demography with data on historical indicators of TB control to parametrise an agent-based model. We use the model to build a rich picture of the current profile of *M.tb* transmission and disease burden in the world’s five highest burden countries in 2016 according to the World Health Organization (WHO): India, Indonesia, China, the Philippines and Pakistan [1].

## Methods

**Fig. 1:**
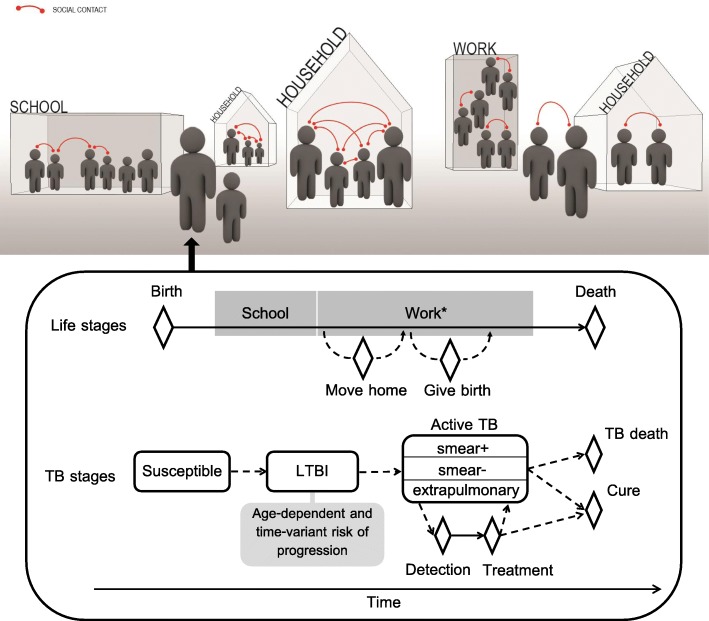
Schematic illustration of the agent-based model. The upper panel represents the structure of the simulated population and the diverse types of contacts simulated (household, school, workplace, other location). The lower panel illustrates individuals’ progression through the various stages of life and infection/disease using diamonds to represent events and boxes for extended phases. Solid arrows indicate deterministic progressions that occur in all surviving individuals, while dashed arrows represent possible but not universal progressions. *Only a fraction of the individuals enters the organised workforce

We developed the SNAP-TB platform (Social Network Abstraction to Profile TB Burden) to simulate *M.tb* transmission and the resulting burden of infection and disease. SNAP-TB is a stochastic agent-based model developed in Python that uses a household, school and workplace framework to generate realistic demographic patterns and social mixing. The population model is overlaid with a TB model that simulates infection, transmission and several existing control measures (Fig. 1). The main model principles are described in the following sections and detailed in Additional file 1, with a description of how data were incorporated in model development and calibration (Additional file 1: Figure S1, and Table S1).

Model initial conditions—replicating the demographic and epidemic configuration of year 2018—were reached by running a burn-in phase to allow demographic processes, age distributions and TB distribution to emerge naturally. The model was then run for five further years to produce outputs. Our approach to model calibration using Latin Hypercube Sampling is described in details in Section 4 of Additional file 1. This approach accounts for uncertainty around 11 important model parameters. Therefore, the results presented in this manuscript are not associated with a single parameter set but emerge from the various parameterisations that were found to produce realistic TB burden according to country-specific data.

### Population model

All individuals are assigned a household at birth. Life events such as forming a couple, moving home and having babies are simulated, such that plausible household compositions emerge from the model. A Siler model is used to derive age-specific natural mortality rates [14], and back-calculated birth rates are used during burn-in to reproduce the desired modern country age distribution.

All children are assumed to attend school (commencing from 3 to 5 years old and completing by 15–21 years old), before optionally entering the workforce, with individuals explicitly assigned to specific schools and workplaces.

### Social mixing

Individuals interact through social contacts that occur in four different contexts: households, schools, workplaces and other locations. A social contact is considered conceptually as either a physical contact or a two-way conversation involving three or more words (consistent with reporting of input data) [9, 15]. All individuals of the same household are assumed to contact each other every day [11, 16]. In contrast, social contacts occurring within congregate settings (schools and workplaces) and in other locations are generated stochastically at each time step. Their frequency and age assortativity pattern are derived for each country from estimates of the location- and age-specific contact matrices [15]. A detailed description of our approach to contact generation in the different settings is provided in Section 2.3 of Additional file 1.

### TB model

Figure 1 illustrates the infection stages simulated. Age-specific parameters derived from empiric data are used to determine whether and when infected individuals progress to active disease [17]. Active cases may be smear-positive, smear-negative or extrapulmonary TB and will either spontaneously cure or die from their disease in the absence of treatment. The type of natural history outcome and the time at which it occurs are randomly generated based on the TB natural history characteristics observed during the pre-chemotherapy era (see Additional file 1, Section 3.1) [18].

*M.tb* transmission may occur when a person with active TB contacts a susceptible individual. The baseline probability that a social contact leads to transmission is calibrated to observed national TB prevalence aggregated for all ages (Additional file 1, Section 4). Empirical estimates of the age-specific TB prevalence (for all countries except India due to absence of data) were compared to model outputs for independent validation but were not used for calibration. We assume that school contacts are less likely to lead to transmission than household contacts (relative risk: RR = 0.89), as are work contacts (RR = 0.82) and other contacts (RR = 0.75). These relative risks are based on the reported proportions of high-intensity contacts by location [9, 10], combined with the assumption that low-intensity contacts are half as likely to lead to transmission as high-intensity contacts (Additional file 1, Section 3.2), with sensitivity analyses used to explore alternative assumptions. The transmission probability also depends on the characteristics of the two individuals making contact, as described in Table 1.

**Table 1:** Model assumptions regarding the factors affecting the risk of transmission

	Modification in risk of transmission	Source
Affecting index infectiousness		
Extrapulmonary TB	Not infectious	–
Smear-negative TB	One quarter as infectious as smear-positive cases	[19, 20]
Age	Infectiousness increases with age (see Additional file 1, Section 3.2)*	[21, 22]
Detection	The transmission probability is halved once the index case has been detected	Assumption
Affecting contact susceptibility		
BCG vaccination	Reduces the risk of the vaccinee becoming infected. Vaccine immunity wanes over time (see Additional file 1, Section 3.2)	[23–25]
Current *M.tb* infection	Reduces the risk of novel infection (RR = 0.21)	[26]

*Assumption explored in sensitivity analysis

The time to detection of active TB is exponentially distributed, and the associated rate is calculated based on the country’s estimated case detection rate (Additional file 1, Section 3.3). Although a detection time is generated for all TB cases, detection only actually occurs if this time precedes the pre-determined time of the natural history outcome.

In our model, all detected cases are commenced on treatment between 0 and 14 days following detection. Successfully treated individuals (i.e. cured or completing treatment) are assumed to clear infection and become susceptible again. If unsuccessfully treated, patients remain active and the TB episode outcome (cure or death) and its timing remain as defined by the TB natural history that was originally generated. Time-variant parameters are used to specify Bacillus Calmette–Guérin (BCG) vaccine coverage, as well as rates of case detection and treatment success. The associated scale-up functions for BCG vaccine coverage and rates of case detection and treatment success are based on WHO data (Additional file 1: Figure S9). The parameters used to inform the model are presented in Table 2.

**Table 2:** Model parameters

Parameter	India	Indonesia	China	Philippines	Pakistan	Source
Demographic						
Simulated population size	20,000	”	”	”	”	
Average household size	4.8	4.0	3.1	4.7	6.8	[27]
Number of schools (/100,000 population)	115	96	37	57	157	[28–32]
Average number of potential contacts at work*	10–30	”	”	”	”	Assumption
Proportion of the adult population engaged in regular work outside of the household (%)	53.8	66.3	68.9	62.3	54.4	[33]
Proportion contacts which are of high intensity by location, with locations listed as households / schools / workplaces / other locations (%)	46 / 30 / 20 / 10	”	”	”	”	[10]
Natural history of TB						
Proportion of active TB cases sm+^a^ / sm−^b^ / extra-p^c^ (%)	50 / 25 / 25	62 / 19 / 19	52 / 24 / 24	60 / 20 / 20	44 / 28 / 28	[34, 35]
Rate of spontaneous clearance (sm+ / closed TB^d^ years^−1^)*	0.18–0.29 / 0.09–0.24	”	”	”	”	[18]
Rate of TB-specific mortality (sm+ / closed TB years^−1^)*	0.33–0.45 / 0.016–0.036	”	”	”	”	[18]
Crude probability of TB transmission during a contact (×10^−4^)*	38.5 (30.2–44.9)	39.8 (34.1–45.2)	36.1 (32.4–40.2)	39.1 (32.3–47.4)	38.3 (30.8–44.3)	Calibrated
Relative probability of transmission per contact if low-intensity contact^e^	0.5	”	”	”	”	Assumption, tested in sensitivity analysis
Programmatic parameters						
BCG vaccine coverage	Time-variant	Time-variant	Time-variant	Time-variant	Time-variant	[36] Additional file 1: Figure S9
Case detection rate	Time-variant	Time-variant	Time-variant	Time-variant	Time-variant	[37] Additional file 1: Figure S9
Time from detection to treatment (days)*	0–14	”	”	”	”	[38–41]
Treatment success rate	Time-variant	Time-variant	Time-variant	Time-variant	Time-variant	[35] Additional file 1: Figure S9

*Parameters included in Latin hypercube sampling; ^a^ smear-positive TB; ^b^ smear-negative TB; ^c^ extrapulmonary TB; ^d^ either smear-negative or extrapulmonary TB; ^e^ reference: high-intensity contact

In order to understand the role played by the past programmatic conditions in shaping the current epidemic picture, we run an additional analysis where all programmatic parameter values are assumed constant and equal to their most recent estimates.

## Results

### Model calibration and validation against age-specific TB prevalence for all forms of TB

**Fig. 2:**
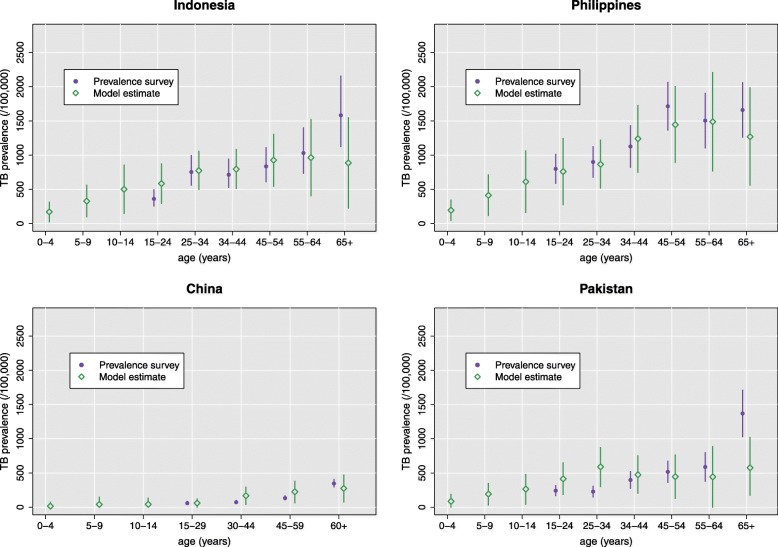
Validation of model outputs against prevalence survey estimates for the age-specific TB prevalence in Indonesia (2014), China (2010), the Philippines (2016) and Pakistan (2011). No data were available for the less than 15-year-old individuals from these surveys. Error bars represent the 95% confidence intervals of the survey estimates (in purple) and the 95% simulation intervals resulting from the stochastic variability of the model and the parameter uncertainty (in green)

The crude probability of transmission per contact obtained from calibration was found to be very similar in each of the five countries, with median values ranging from 0.00361 in China to 0.00398 in Indonesia (Table 2 and Additional file 1: Figure S10). We validated the model by comparing the resulting age-specific prevalence estimates to those obtained from the prevalence surveys conducted in Indonesia (in 2014), China (in 2010), the Philippines (in 2016) and Pakistan (in 2011) (Fig. 2). Age-specific estimates of prevalence are also presented for India (Additional file 1: Figure S11), although comparison to data was impossible in the absence of a prevalence survey.

### Profile of *M.tb* transmission

**Fig. 3:**
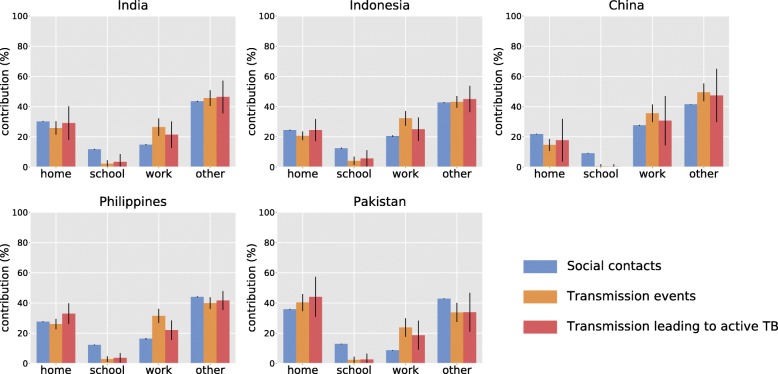
Contributions of the various locations to the burden of contact and transmission. Error bars represent the 95% simulation intervals

In order to better characterise transmission, we recorded contacts’ characteristics by tracking their location (school, work, home, other) and the age of the individuals involved. The same information was recorded for each transmission event by location, along with whether the associated infection resulted in active TB (Fig. 3). According to our model, contacts occurring in locations other than home, school or workplace are a major driver of *M.tb* transmission in each of the five countries, with contributions ranging from 34% (28–40, 95% simulation interval) of the total number of transmission events in Pakistan to 49% (44–55) in China. Household contacts were estimated to be the predominant driver of *M.tb* transmission in Pakistan (40%, 35–46). The estimated proportion of active TB burden attributable to household contacts varies between 17% (4–32) in China and 44% (31–57) in Pakistan.

**Fig. 4:**
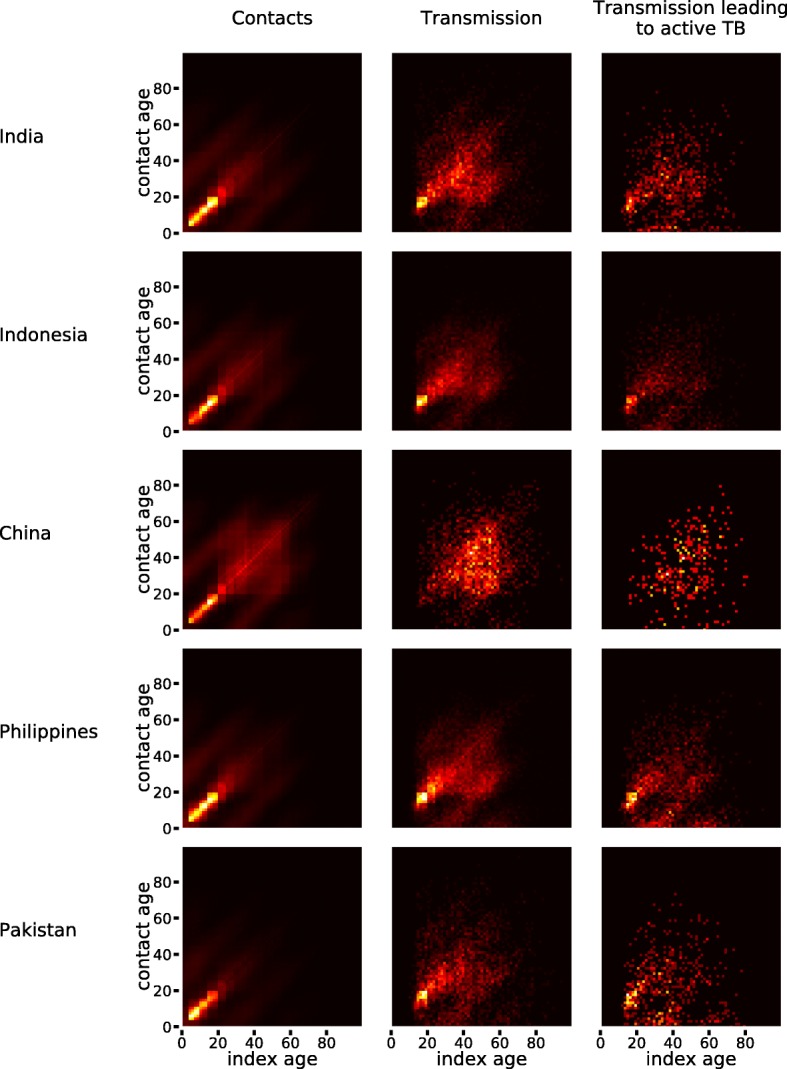
Age-specific pattern of social mixing and transmission

Figure 4 presents the age-specific contact and transmission patterns obtained from simulation. Contact patterns disaggregated by location are presented in Additional file 1: Figure S5. We note that our approach of allowing for household compositions to emerge naturally during simulation led to plausible age-specific contact patterns that are similar to those described in several social mixing studies [9–11]. The high-intensity contact zones naturally translate into high densities of *M.tb* transmission, except where index individuals are aged under 15 years (due to lack of infectiousness) and where contact recipients are young and therefore retain immunity from BCG vaccination. In contrast, the effect of immunity from infection was no longer observed when considering only contacts leading to active disease. This finding is due to the fact that young individuals are at higher risk of progression to active disease than adults [42, 43]. Our results highlight that the 15–19 years age category represents a critical driver of transmission in all countries except China. In India, Indonesia, the Philippines and Pakistan, we estimate that more than 20% of transmission events involve in this age category as either index or recipient (Table 3).

**Table 3:** Contributions of the 15–19-year-old individuals to the estimated total number of transmission events between 2018 and 2022

Estimated proportion of transmission events for which	The index is 15–19 y.o.	The recipient is 15–19 y.o.	Both individuals are 15–19 y.o.	At least one 15–19 y.o. is involved
India				
All transmissions	11%	14%	5%	20%
Transmissions leading to TB	13%	13%	5%	21%
Indonesia				
All transmissions	14%	15%	7%	22%
Transmissions leading to TB	16%	14%	7%	23%
China				
All transmissions	2%	5%	1%	7%
Transmissions leading to TB	2%	6%	1%	7%
The Philippines				
All transmissions	14%	16%	7%	23%
Transmissions leading to TB	15%	14%	6%	23%
Pakistan				
All transmissions	16%	16%	7%	25%
Transmissions leading to TB	17%	14%	7%	25%

### Age distribution and risk associated with the current latency reservoir

**Fig. 5:**
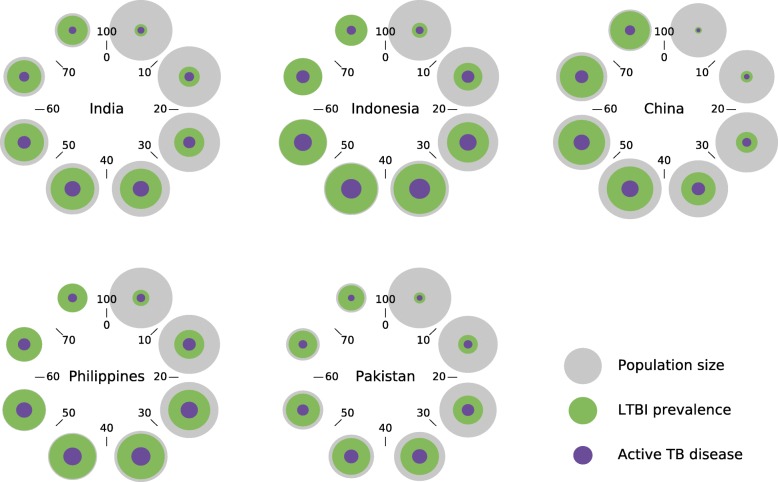
Age distribution of latent tuberculosis infection. Coloured discs should be interpreted as spheres (to increase the relative size of the smaller spheres), with the volume of the spheres being proportional to the following quantities: 2018 total population (grey), size of the LTBI pool in 2018 (green), and number of individuals currently infected in 2018 who will ever develop active TB (purple). The numbers surrounding each disc indicate the age categories represented. Note that LTBI prevalence is predicted to reach extremely high levels among the oldest age category, which is explained by the high historical intensity of transmission in these countries and by the fact that we do not incorporate LTBI clearance

We estimated the country-level prevalence of LTBI in 2018 at 25% (14–36), 47% (35–55), 30% (18–41), 43% (34–52) and 25% (14–39) in India, Indonesia, China, the Philippines and Pakistan, respectively. These estimates are very similar to those obtained from a previous modelling work, and a comparison between the two studies is presented in Additional file 1: Figure S12 [4]. Figure 5 presents the age-specific size of the LTBI reservoir as estimated for 2018 (green spheres), as well as the risk that it represents in terms of future TB disease (purple spheres). The relative LTBI prevalence steadily increases with age in all countries, whereas the absolute LTBI burden decreases at advanced ages due to population mortality.

According to our model, the age category that represents the highest risk in terms of future disease emanating from current infections is the “30–39-year-old” age category in India (29% of future TB burden), Indonesia (27%), the Philippines (26%) and Pakistan (31%), while the “40–49-year-old” age category was most prominent in China (33%). The youngest age category “0–9-year-old” includes very few infected individuals in all countries, although the per-infection risk of disease is much higher in this age category than in older populations.

### Age profile of active TB

**Fig. 6:**
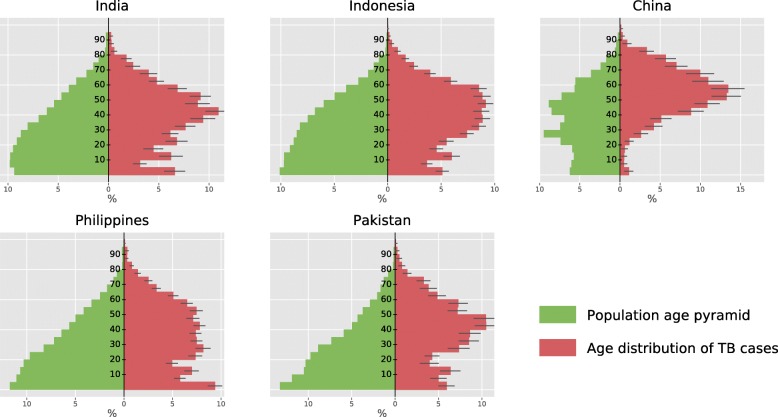
Age distribution of TB cases. The population age distribution (green) was captured at the starting time of analysis (year 2018). Age of TB cases at activation (red) was recorded over a period of 5 years starting from 2018. Error bars represent the 95% simulation intervals obtained for the TB age distribution

Figure 6 shows the estimated age distribution of TB cases in the five countries. In China, we estimate that TB affects the ≥ 45-year-old category much more severely than the younger age categories, accounting for 76% (73–79) of the national TB burden. In particular, the age category 55–59 is the most represented, alone contributing 13% (11–15) to the Chinese TB burden.

Young individuals (particularly those aged under 5 or 10–14) are severely affected with active TB in India, Indonesia, the Philippines and Pakistan. Although the Philippines and Pakistan present similar population pyramids, their TB age distributions differ noticeably. We find a prominent peak for the 40–49-year age category in Pakistan (contributing 21% of TB burden), which is not observed in the Philippines (14% for the same age category). In contrast, young adults (aged 20–24 years) constitute a considerably larger proportion of the burden in the Philippines (7%) than in Pakistan (4%). Finally, the youngest age category (0–4-year-old) was estimated to be a major contributor to the TB epidemic in the Philippines, with an estimated contribution reaching 9% (9–10). The proportion of paediatric TB (< 15 years old) among all TB cases is estimated at 17%, 15%, 2%, 22% and 18% in India, Indonesia, China, the Philippines and Pakistan, respectively.

Additional file 1: Figure S17 presents the TB age distribution obtained for the Philippines in the sensitivity analysis assuming constant historical programmatic conditions (i.e. removing time-variant programmatic parameters). We note that a substantial share of the estimated TB burden is shifted towards the youngest age categories under this scenario, making the TB age profile more similar to the population age distribution which is highly inconsistent with the 2016 prevalence survey results. In another sensitivity analysis where we assume that low-intensity contacts cannot result in transmission, households become the predominant context of *M.tb* transmission in all countries except China (Additional file 1: Figure S18). We observe that the calibrated crude probability of transmission per contact remains similar across each of the five countries, regardless of the assumption made around the relative risk of transmission through low-intensity contacts as compared to high-intensity contacts (Additional file 1: Figure S26).

## Discussion

We present a detailed representation of *M.tb* transmission and the resulting burden of infection and TB disease in the five highest TB burden countries. Using an agent-based model that combines household structure, social mixing matrices, age-specific infectiousness and reactivation rates, and the history of national TB control, we provide insights into major TB epidemic characteristics that would be otherwise unattainable. These include the age profile of *M.tb* transmission, the age-specific LTBI prevalence and associated risk of future disease, the age distribution of incident TB cases, and the contributions of different contact types to the burden of transmission and disease. Furthermore, we demonstrate that the demographic and programmatic model inputs alone are sufficient to explain the considerable heterogeneity in burden observed between countries, with calibrated per-contact transmission rates being very similar.

We show that the 15–19-year-old age category is a major driver of *M.tb* transmission in all countries except China. This observation, which is due to the high frequency of contacts and waning of immunity conferred by BCG at this age [9, 24], contrasts with the relatively low estimated burden of active disease observed in this age group. This finding highlights the marked difference between the age profile of *M.tb* transmission and that of TB burden and implies that relying only on the observed burden of active disease to understand the age profile of a TB epidemic would provide an incomplete and misleading picture. The relatively low TB burden estimated in the 15–19-year-old age group may explain why adolescents and young adults constitute a neglected group in global TB control and are rarely considered as a target population for preventive measures [44]. However, our model suggests that preventing infection (e.g. by raising TB awareness) and reactivation (through prophylaxis treatment) within this group could potentially yield significant burden reductions in the older age categories. Identifying individuals that should be targeted with TB prevention is critical to guide control policies, as world political leaders have recently declared their commitment to provide 30 million people with preventive treatment by 2022 [45].

Another age-specific transmission peak was identified between parents and their children in all settings, which is especially concerning for children under five, as they are more likely to progress to active disease once infected [42]. This observation underscores the critical importance of implementing rapid screening and control measures for the youngest contacts of identified adult pulmonary TB cases. We estimate that childhood TB (< 15 years old) contributes to around one fifth of the total TB incidence in India, the Philippines and Pakistan, as a consequence of the countries’ young populations and their high contact intensities. This is in line with previous estimates obtained in other high-incidence settings [21, 22]. Incorporating age-specific epidemiological characteristics such as infectiousness, risk of activation and waning BCG immunity allowed us to further refine the distribution of TB cases among < 15-year-olds using 5-year age brackets. This insight is particularly valuable because it is difficult to directly assess in real-world settings due to the challenges encountered with the diagnosis and surveillance of paediatric TB [22].

The TB age profile in China is dramatically different to that reported for the other four countries modelled in this study. China experiences TB principally in the oldest part of the population, with three quarters of the TB burden attributed to the ≥ 45-year-old category, although population ageing is not the only explanation for this phenomenon. The dramatic improvement in case detection since 2000 combined with high treatment success rates (over 90%) maintained over the last three decades has resulted in a dramatic fall in *M.tb* transmission over recent years, such that younger cohorts have now been much less exposed to the pathogen than preceding generations. This suggests that the current burden of active TB in China results primarily from reactivation of old infections that were acquired when transmission was still intense, consistent with previous work [46]. The importance of the programmatic history in shaping the current age profile of TB was further highlighted by the discrepancies observed in our sensitivity analysis performed without time-variant parameters and ignoring past TB control.

We provide estimates of the age-specific size of the LTBI reservoir, along with the risk that it represents in terms of future disease. Knowing who is latently infected provides valuable knowledge for policy-makers when designing contextualised preventive strategies. Our country-specific predictions could be used to estimate the yield of mass LTBI screening/treatment programs targeted at specific age categories, both in terms of the number of current infections treated and future disease episodes prevented. Although broad recommendations for the management of LTBI have been adopted [44], little is known about how best to adapt these to local programmatic and epidemiological contexts.

Social interactions occurring outside of homes, schools and workplaces were identified as the main driver of transmission in India, Indonesia, China and the Philippines. This finding implies that control measures focusing on close and easy-to-identify contacts of diagnosed TB cases may have a limited impact at the population level in these settings. This is consistent with other modelling works which suggest a limited role of household transmission due to contact saturation [12, 13]. In contrast, simulated *M.tb* transmission in Pakistan occurs primarily in homes due to Pakistan’s large average household size (6.8 persons). Therefore, interventions such as providing household contacts with screening and prophylaxis treatment are likely to be more efficient in Pakistan. We found that the contribution to the TB burden from household contacts and those occurring in “other locations” was sensitive to our assumptions about the relative risk of transmission through low-intensity contacts as compared to high-intensity contacts. However, it is important to note that the two scenarios considered in our sensitivity analyses are extreme and likely unrealistic, as they represented either a null risk of transmission for low-intensity contacts or a risk that is equal to that of high-intensity contacts.

The transmission probability, calibrated separately to the different TB burdens, was remarkably similar in the five countries we studied, providing confidence about model robustness. Moreover, it indicates that the socio-demographic characteristics included, along with the simulated time-variant programmatic changes, are able to account for the bulk of the heterogeneity in TB burden. This finding also suggests that the per-contact risk of transmission could be similar in all settings after adjustment for age, household composition and other factors relevant to infectiousness and susceptibility. The validity of our model was further reinforced by the closely matching estimates obtained when comparing our simulated age-specific prevalence to the equivalent estimates from the prevalence surveys conducted in the Philippines, Indonesia, China and Pakistan. Furthermore, our estimates of LTBI prevalence were remarkably close to those produced in a previous modelling study [4], although our 95% simulation intervals are much wider than those obtained in the previous work.

A limitation of this study is that the social mixing matrices that we incorporated into the model were not directly obtained from contact surveys. Instead, we used country-specific estimates generated by combining survey data from other countries with an extrapolation model [15]. Our estimates will therefore be refined further as local mixing data such as those provided by the POLYMOD study became available for a greater range of contexts [9]. Another potential limitation is that we opted for model parsimony in relation to factors including gender, comorbidities and sub-national geography, which are the subjects of current work. Due to high computational expense, we were unable to employ classic approaches such as Monte-Carlo Markov Chain methods to perform uncertainty analysis. However, the parameter values used in the model are based on empirical evidence and official reports, which has dramatically reduced the need to make assumptions. Furthermore, multi-dimensional uncertainty was included around 11 input parameters in order to explore various model configurations and selected parameters considered the most likely to affect model outputs were varied in sensitivity analyses, which did not jeopardise our main findings.

## Conclusions

We show that it is possible to create new and valuable insights into the profile of local TB epidemics by combining agent-based simulation with social mixing data and TB control history. We demonstrate that social contacts involving 15–19-year-old individuals are a critical driver of TB which is not evident from the age distribution of TB cases. Our model also highlights the high burden of childhood TB in high-incidence settings and underlines the critical role played by parents-to-children transmission.

## Supplementary information

**Additional file 1.** Technical appendix providing detailed descriptions of the methods and results of sensitivity analyses.

## Data Availability

All data generated or analysed during this study are included in this published article and its supplementary information file.
