# Supplementary material for: Profiling Mycobacterium tuberculosis transmission and the resulting disease burden in the five highest tuberculosis burden countries
Source: BMC Med. 2019 Nov 22;17:208. doi: 10.1186/s12916-019-1452-0 (PMC6873722; doi:10.1186/s12916-019-1452-0)
Supplement: Supplementary file 1 — Additional file 1. Technical appendix providing detailed descriptions of the methods and results of sensitivity analyses. [file 12916_2019_1452_MOESM1_ESM.pdf]

Profiling *Mycobacterium tuberculosis* transmission and the resulting disease burden in the five highest tuberculosis burden countries

Additional file 1

Romain Ragonnet, James M. Trauer, Nicholas Geard,

Nick Scott, Emma S. McBryde

Contents

1 General description of the agent-based model 4
2 Demographic structure and contacts between individuals 6
2.1 Household structure, birth and death processes 6
2.2 Schools and workplaces 8
2.3 Social contacts 9
2.3.1 Definition of a social contact and general approach to contact generation 9
2.3.2 Household contacts 9
2.3.3 School and workplace contacts 9
2.3.4 Contacts in other locations 11
2.3.5 Simulated overall contact patterns 11
3 Model of tuberculosis 12
3.1 Natural history 12
3.2 TB transmission 13
3.3 Detection and treatment of active TB 15
3.3.1 Case detection rate (proportion) 15
3.3.2 Calculation of the rate of the exponential process associated with the time to detection 15
3.3.3 Treatment of active TB 15
3.4 Time-variant programmatic parameters 15
4 Model calibration 17
4.1 Description 17
4.2 Calibration targets 18
4.3 Posterior parameter ranges 18
5 Additional results 20
5.1 Estimated age-specific active TB prevalence for India 20
5.2 Estimated prevalence of latent TB infection 20
5.3 Considering an alternative age-specific infectiousness profile 21
5.4 Ignoring the past programmatic background 25
5.5 Alternative scenarios for the risk of transmission through low- versus high-intensity contacts 25
5.5.1 Only high-intensity contacts can result in transmission (SA 1) 25
5.5.2 Equal risk of transmission for low- and high-intensity contacts (SA 2) 29
5.5.3 Findings from sensitivity analyses SA 1 and SA 2 31
6 References 33

List of Figures

Figure S1. Overall description of the relationships between the data and the model. 5
Figure S2. Siler model survival function fitted to data from country life-tables. 7
Figure S3. Distribution of the household sizes emerging during simulation. 8
Figure S4. Age-preference functions used to calculate the probabilities of contact within workplaces and schools. 10
Figure S5. Simulated age-specific contact patterns by country and location... 12
Figure S6. Assumed profiles of infectiousness over age. 14
Figure S7. Assumed wane profile of BCG efficacy... 14
Figure S8. Fitting of time-variant programmatic parameters to data. 16
Figure S9. Posterior parameter ranges associated with the 100 calibrated simulations per country.................... 19
Figure S10. Age-specific active TB prevalence for India in 2015 20
Figure S11. Estimated LTBI prevalence according to our model versus *Houben & Dodd 2016*. 20
Figure S12. Alternative age-specific infectiousness profile for sensitivity analysis 21
Figure S13. Contributions of the various locations to the burden of contact and transmission (alternative infectiousness profile).. 22
Figure S14. Age-specific profile of social mixing and transmission (alternative infectiousness profile)............ 23
Figure S15. Age-distribution of latent tuberculosis infection (alternative infectiousness profile) 24
Figure S16. Age-distribution of TB cases (alternative infectiousness profile) 24
Figure S17. Results for the Philippines when no time-variant parameters are included.................................... 25
Figure S18. Contributions of the various locations to the burden of contact and transmission (scenario SA 1) . 26
Figure S19. Age-specific profile of social mixing and transmission (scenario SA 1) 27
Figure S20. Age-distribution of latent tuberculosis infection (scenario SA 1) 28
Figure S21. Age-distribution of TB cases (scenario SA 1)... 28
Figure S22. Contributions of the various locations to the burden of contact and transmission (scenario SA 2) . 29
Figure S23. Age-specific profile of social mixing and transmission (scenario SA 2) 30
Figure S24. Age-distribution of latent tuberculosis infection (scenario SA 2) 31
Figure S25. Age-distribution of TB cases (scenario SA 2)... 31
Figure S26. Calibrated probabilities of transmission per contact for sensitivity analyses.................................. 32

List of Tables

Table S1. Description of data sources and incorporation in the model. 5
Table S2. Estimated values of the Siler model parameters... 7
Table S3. Estimated parameter values for the dispersion parameters σ associated with workplace contacts. 10
Table S4. Parameters used to characterise the natural history of TB.. 13
Table S5. Uncertainty parameters and their prior distributions. 17
Table S6. Estimates of TB prevalence used for calibration. 18

1 General description of the agent-based model

This document describes our approach to developing an agent-based model that simulates a dynamic population of individuals who each have their own characteristics in terms of demographics and disease status. These individuals interact with one another and social contacts between them are explicitly simulated, allowing for transmission of *Mycobacterium tuberculosis* (*M. tb*) to occur and to be fully traced. Individuals are assigned to households and may attend schools or workplaces, which allows for implementation of various types and frequencies of contacts between them. This framework also makes possible the simulation of TB-specific control interventions. For example, contact tracing following diagnosis of an active tuberculosis (TB) case or blanket preventive treatment within a school of a workplace could easily be explored.

We simulate a population of 20,000 individuals whose actions and interactions are computed through an iterative process, in which a time-step represents a given duration (seven days in this study). The population size was determined so that the number of individuals simulated is sufficiently high to be considered as a representative “sample” of a country population, while retaining reasonable computation times. At each iteration, the demographic and TB-specific characteristics of the individuals are updated through processes that pertain to natural transitions (ageing, death, birth and starting/finishing school or work) and disease-related events (transmission, activation, detection, treatment and TB-related death). Most of these processes are stochastic, such that running multiple simulations with an identical parameter set will result in different outcomes. For each country, 100 independent simulations (selected during calibration, see Section 4) are run and the mean and central 95% credible interval are reported for each model output.

Before running the simulations associated with the results presented in the main text, we obtained model initial conditions by performing a burn-in to allow demographic processes, age distributions and TB distribution to emerge naturally. This burn-in phase runs for 200 years in total, with TB only introduced after the first 100 years of simulation. The model is run for a further period of five years during which model outputs are recorded. One full simulation (simulating 205 years) is completed in about three hours using an Intel Xeon E3-12xx v2 processor (3.1 GHz, 8MB Cache).

Figure S1 provides an overall description of the relationships between the model and the different types of data and Table S1 describes the data sources and how the data are used to inform the model.

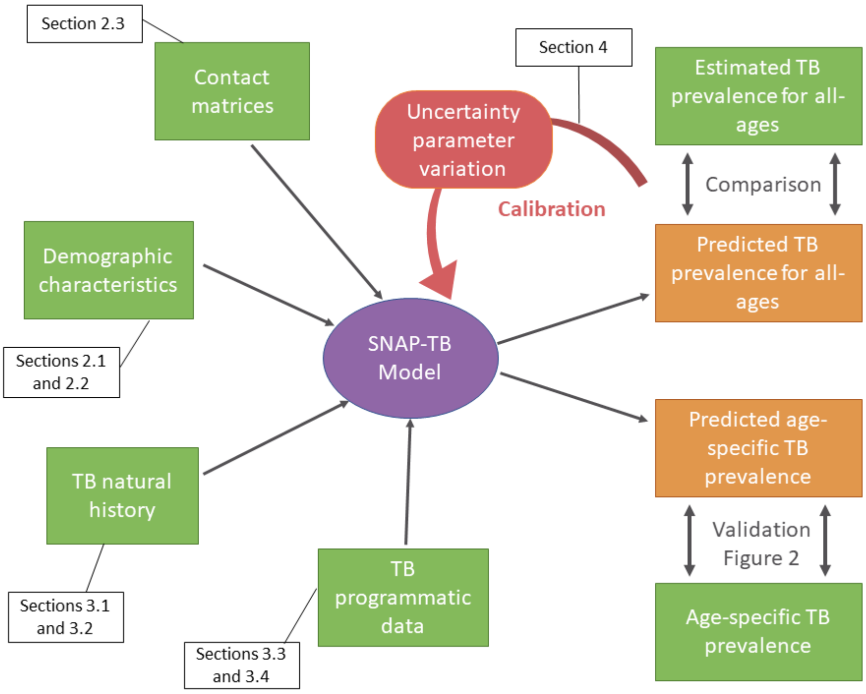

Figure S1. Overall description of the relationships between the data and the model.
The green rectangles represent the different types of data that are used either as direct model inputs (contact matrices, demographics, TB natural history and TB programmatic data) or for the purpose of model calibration (TB prevalence for all-ages) or model validation (age-specific TB prevalence). The model outputs used for calibration and validation are represented in orange and the parameter fitted through calibration is represented in red. The sections of this document that present the different components represented in this diagram are indicated in black boxes.

Type of data	Sources	How this data is used in the model	Described in
Contact matrices	Country-specific and age-specific estimates of contact rates by setting.[1]	The contact rates are used as **inputs** to generate social contacts in schools, workplaces, and the “other locations” settings.	Section 2.3
Demographic characteristics	Country-specific data from the United Nations database,[2] governmental websites,[3-6] and The World Bank.[7,8]	Used as **inputs** to parameterise the average household size, the number of schools, and the labour force participation.	Sections 2.1 and 2.2
TB natural history	Dynamics of TB reactivation.[9-11] Literature on TB patients’ infectiousness. [12-14] Systematic review from the pre-chemotherapy era on TB disease duration.[15]	These model **inputs** define the history of infection and disease in absence of treatment.	Sections 3.1 and 3.2
TB programmatic data	Literature on BCG vaccine efficacy.[16,17] WHO reports for BCG coverage, case detection rates, and treatment success rates.[18-20]	Used as time-variant **input** parameters to define the probability of being vaccinated at birth, the time to detection, and the probability of treatment success.	Sections 3.3 and 3.4
TB prevalence for all-ages	National TB reports and WHO reports. See Section 0 for details.	Used as target for **model calibration**.	Section 4
Age-specific TB prevalence	Prevalence surveys for all countries except India.	Used for comparison as independent **model validation**.	Figure 2 (main text)

Table S1. Description of data sources and incorporation in the model.

2 Demographic structure and contacts between individuals

2.1 Household structure, birth and death processes

A number of households (obtained by dividing the total population by the average household size) is created to host the individuals. Household creations or removals may also occur as the total population size varies during simulation in order to maintain the country average household size. At birth, individuals are allocated to households which they may or may not leave as they enter adulthood. Individuals ‘enter adulthood’ and become eligible to leave their original home at an age which is generated at random from a uniform distribution between 18 and 30 years old (could be adjusted for other model applications). If an empty household is available at the time that an individual enters adulthood, they move to the available household and form a couple with an individual who also entered adulthood within the last 12 months. This household then becomes eligible to receive newborn individuals for a period of ten years. If an individual is not selected to enter an empty household within one year of entering adulthood (the number of places available in empty households being fewer than the number of new adults), they remain in their original household for the remainder of their life. However, they may still procreate in their original household (and without living with a partner), which similarly becomes eligible to receive newborns for ten years. Households are not permitted to receive two successive newborns within the same twelvemonth period, in order to maintain realistic age differences between siblings.

The age at which individuals die is randomly generated from a Siler model. This model is defined by an age-dependent hazard function that defines the force of mortality:

$$h(x) = e^{\alpha_1 - \beta_1 x} + e^{\alpha_2 + \beta_2 x} + e^{\alpha_3}$$

where x represents the individual’s age. α_1, α_2 and α_3 are scale parameters which reflect the level of mortality at younger ages, older ages, and overall respectively. β_1 and β_2 are age-trend parameters describing the slope of the hazard trajectory at younger ages and older ages.

We fit the associated survival function (obtained after calculation)

$$S(x) = \exp\left[-\int_0^x h(a).da\right] = \exp\left[\frac{e^{\alpha_1}}{\beta_1}\left(e^{-\beta_1 x} - 1\right) - \frac{e^{\alpha_2}}{\beta_2}\left(e^{\beta_2 x} - 1\right) - e^{\alpha_3} x\right]$$

to real-world survival data obtained from the country-level life tables published by WHO[21] (Figure S2). Fitting is achieved by sum of squares minimisation. Table S2 presents the values of the Siler model parameters obtained for the five different countries.

The life duration density function corresponding to the Siler model is obtained by multiplying $S(x)$ by $h(x)$. Finally, the resulting density function is used in the model to sample ages at death using rejection sampling.

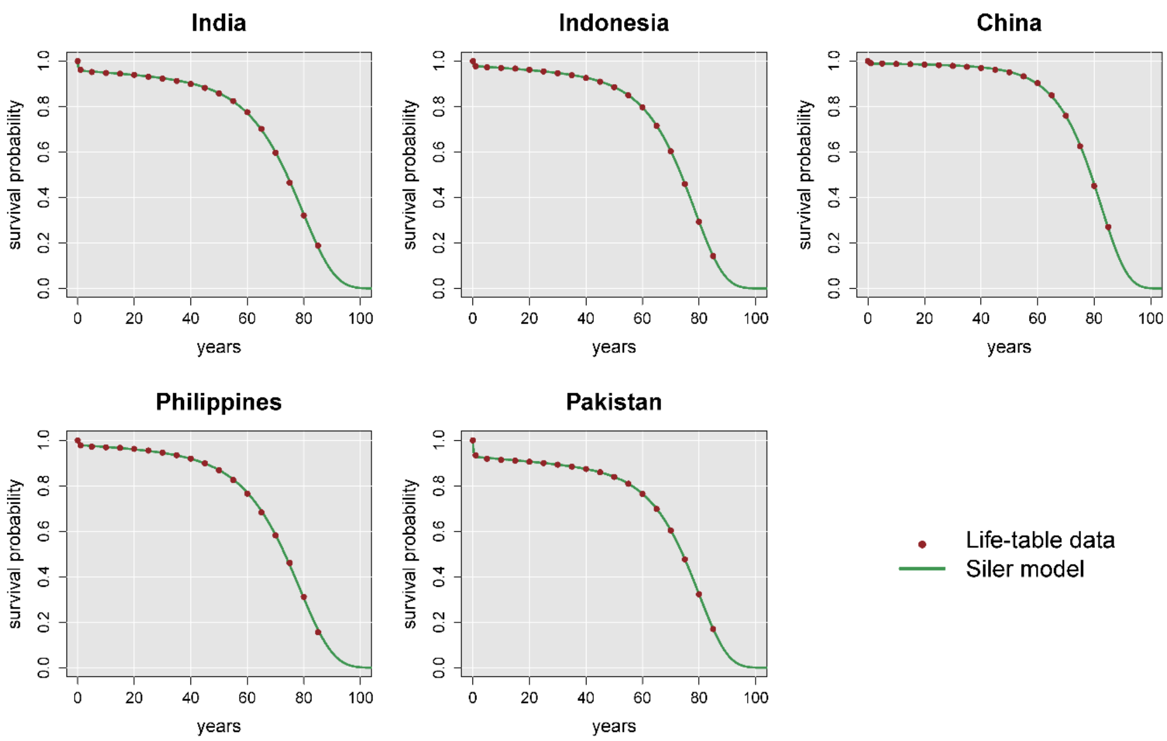

Figure S2. Siler model survival function fitted to data from country life-tables.

	α_1	β_1	α_2	β_2	α_3
India	-2.43	4.02	-10.06	0.10	-7.12
Indonesia	-1.76	4.00	-9.58	0.09	-7.08
China	-3.15	4.04	-11.25	0.11	-8.51
Philippines	-2.47	4.02	-9.25	0.09	-7.19
Pakistan	-1.20	3.98	-10.38	0.10	-6.84

Table S2. Estimated values of the Siler model parameters.

Three different fertility scenarios are implemented and used sequentially during each phase of simulation. First, fertility replacement is assumed during the first (100 year) phase of burn-in, introducing a newborn into the population every time that an individual dies. Next, a variable birth rate is used during the second (100 year) burn-in phase, in order to reach a targeted age-distribution (country age-pyramid) at the commencement of the analysis phase, as follows. Let τ denote the time remaining before burn-in completion ($0 \leq \tau \leq 100$ years). The number of births occurring at time $t = 200 - \tau$ is calculated by dividing the desired number of individuals of age τ that should be alive at the end of the burn-in phase by the probability of survival after τ years obtained from the Siler mortality model. Finally, once burn-in is completed, a constant rate equal to the inverse of the mean life expectancy is used to generate births during the analysis phase (of five years).

For each birth simulated, a newborn is allocated to an eligible household (see description of eligible households in the first paragraph of this section). The household receiving a newborn is selected using a probabilistic approach that favours smaller households. Let s_i denote the size of the eligible household i. The newborn's

household is chosen using a multinomial distribution where the eligible household i is selected with probability $\frac{\gamma}{s_i}$, where γ is a normalising constant (i.e. $\gamma = \frac{1}{\sum_i 1/s_i}$).

Figure S3 shows the household size distributions from ten simulations of 50,000 individuals for the five different countries analysed. These outcomes emerge naturally according to the input average household size and from the process described in the above paragraph.

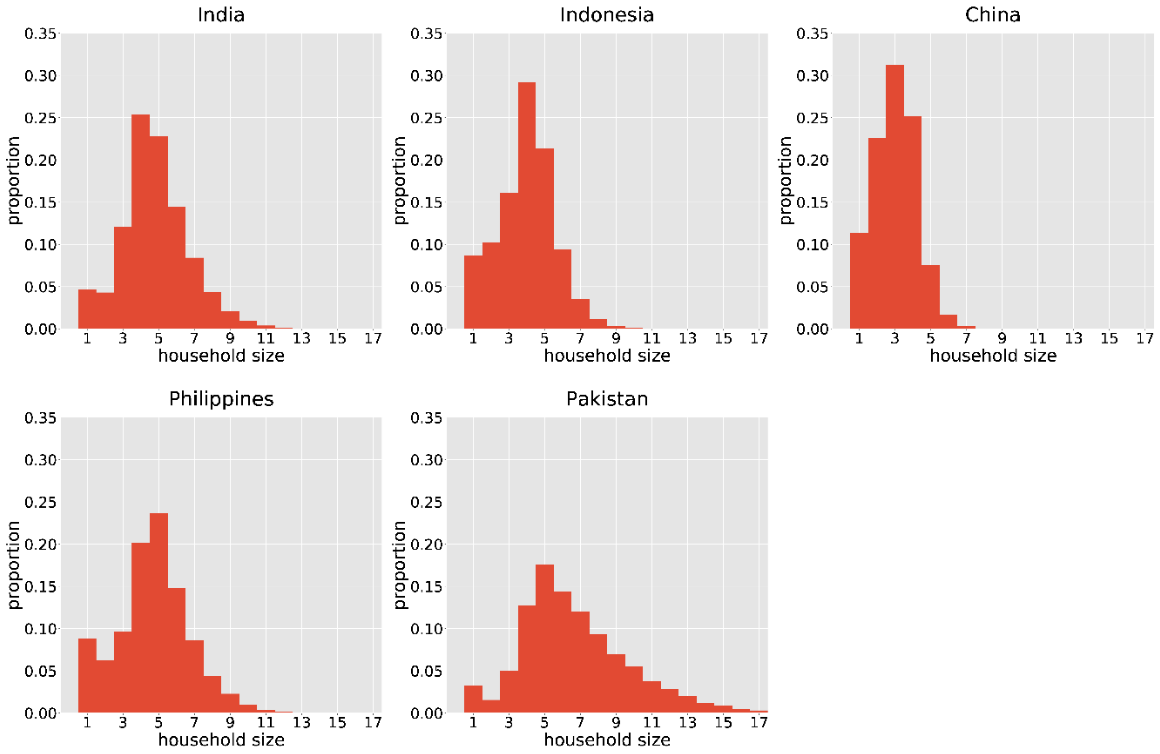

Figure S3. Distribution of the household sizes emerging during simulation.

2.2 Schools and workplaces

The population model described is overlayed with a framework of schools and workplaces. We assume that each household is associated with a single school, such that children who live in the same household necessarily attend the same school. Schools are randomly assigned to each household during model initialisation. Every "school-aged" individual is considered to attend school. A school-age range is drawn for each individual using uniform distributions for the starting age (3-5 years old) and the leaving age (15-21 years old). When individuals complete school, they may become active workforce participants (and are then randomly assigned to a workplace), and otherwise will never engage in employment outside of the household.

In the context of this study, a workplace is intended to capture a group of individuals who are working at the same location and between whom the intensity of contact in their professional context is sufficient to allow for *M.tb* transmission. That is, the term "workplace" may actually refer to a subsection or department of a company, or a subgroup of individuals who are working together on a regular basis rather than a whole corporation. This entity is characterised by its average size, which is assumed to be the same in each country (Table 2, main text) in the absence of country-specific data to inform this quantity. The number of workplaces is calculated by

dividing the number of working individuals in the population by the average workplace size. When individuals enter active employment, they are randomly assigned to a workplace in which they remain until retirement. Each individual's retirement age is randomly drawn from a uniform distribution in the 55-70 age range.

Schools and workplaces may be created or removed during simulation as the number of households varies with variation in the total population size.

2.3 Social contacts

2.3.1 Definition of a social contact and general approach to contact generation

We define a social contact between two individuals as either a physical contact or a two-way conversation involving three or more words, because this definition was used in the studies from which the contact matrices were extracted [1,22]. Social contacts are recorded on a daily basis, such that for each index individual, we record a list of contacted individuals along with the number of times (days) that interactions occurred. Social contacts are classified into four categories according to the context in which they occur: household, school, workplace and other locations. Note that social contact generation is only performed for individuals who are infectious (unless specifically requested for the purpose of illustration, e.g. to generate Figure S5).

2.3.2 Household contacts

In our model, all individuals of the same household contact one another every day. While contact patterns between household members are likely to be more complex in the real world, two recent studies support the use of within-household homogenous mixing assumptions for epidemic modelling [23,24].

2.3.3 School and workplace contacts

We use a similar approach for both schools and workplaces to the generation of social contacts between individuals. Each day, a contact is generated between individuals a and b with probability $P_c(a,b) = p\,.\,f_\sigma(a,b)$ where f_σ is an age-preference multiplier function which depends on the age difference between individuals a and b and p represents the probability that two individuals within the same structure and of the same age contact one another during a typical day. Our approach to determining f_σ and p is described in the following section.

The age-preference function for contact between individuals is simulated using a Gaussian function $f_\sigma(x) = e^{\frac{-x^2}{2\sigma^2}}$, where x is the age difference between two individuals and σ is a dispersion parameter. f_σ represents the relative likelihood that two individuals contact each other given their age-difference as compared to two individuals of same age. Due to lack of available data, we could not estimate country-specific values for the parameter σ associated with schools. Specifically, five-year age categories were used to report the contact matrices that inform our model,[1] which is too broad to allow for any preference profile to be extracted within school ages alone. We therefore assume a constant value $\sigma = 2$ years for all countries, which is equivalent to saying that 68% of within-school contacts involve individuals with an age gap <2 years. This intended to reflect significant cohorting by single year of age due to school classes being largely comprised of children born in the same twelve month period, while also allowing for significant mixing outside of the classroom. In contrast, the parameter value associated with workplaces is estimated from the workplace-specific contact matrices reported by Prem et al. [1]. These matrices provide the age-specific daily contact rates between individuals using five-year

age groups. Let $(A_{i,j})$ denote one of these workplace contact matrices, where i and j are the different age group indices. An estimate of σ is obtained by minimising the following distance:

$$d_\sigma = \sum_i \sum_j \left\{ \frac{A_{i,j}}{A_{i,i}} - \frac{P_j}{P_i} f_\sigma(5\,|j-i|) \right\}^2 ,$$

where we only sum over the indices i and j that are associated with working age age-groups and where $A_{i,i}, P_i \neq 0$ (always verified in practice). The term $\frac{A_{i,j}}{A_{i,i}}$ corresponds to the relative contact rate between an individual from group i and an individual from group j, as compared to the contact rate between two individuals form group i. The distance d_σ compares these quantities to their associated measure under our model assumption, which is the Gaussian function f_σ evaluated for $x = 5\,|j-i|$, the average age-difference between group i and group j. Note that this quantity is adjusted by multiplying with the age-distribution ratio $\frac{P_j}{P_i}$ where P_i represents the proportion of the population belonging to age-category i. This approach allows us to isolate the behavioural age-preference component of age-assortativity from the effect of the background age-distribution which affects the absolute reported age-specific contact rate values.

Figure S4 presents the age-preference profiles obtained for the five different countries considered in our analysis and Table S3 reports the estimated values of σ corresponding to workplace contacts.

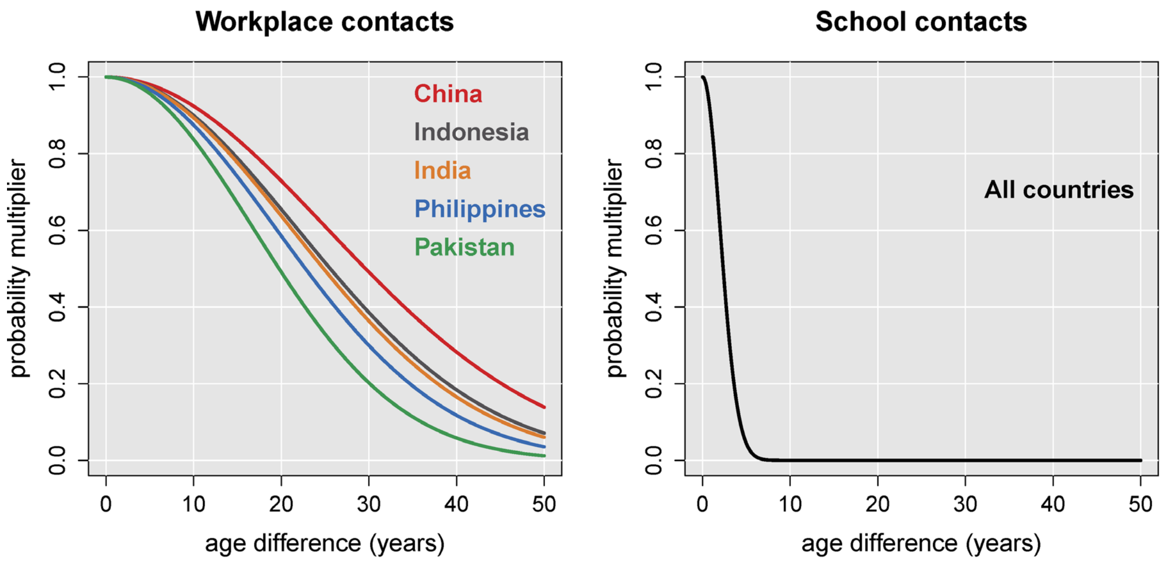

Figure S4. Age-preference functions used to calculate the probabilities of contact within workplaces and schools.

	India	Indonesia	China	Philippines	Pakistan
σ	21.1	21.7	25.2	19.3	16.8

Table S3. Estimated parameter values for the dispersion parameters σ associated with workplace contacts.

To estimate p(the probability of contact with an age-matched person in the individual's school or workplace) for each index individual, we extract the total number of contacts N expected to occur during one day within the school or workplace from the contact rate matrices.[1] This number should be equal to the sum of all the individual probabilities of contact with the other members of the school or workplace, because it is Poisson binomial distributed. That is, $N = \sum_b P_C(a,b) = p \sum_b f_\sigma(x_{a,b})$ where a designates the index individual and b the other members of the congregate setting. Given that f_σ has been previously estimated (see preceding paragraph), we obtain p using the following equation: $p = N/\sum_b f_\sigma(x_{a,b})$.

2.3.4 Contacts in other locations

Prem and colleagues also provided age-specific contact rates for social interactions occurring outside of households, schools and workplaces [1]. We use these estimates to generate such social contacts in the model. For a given index individual belonging to age category i, we generate the numbers of contacts for all the different age categories j using Poisson distributed variables. The mean of each of these Poisson variables is the age-specific contact rate $A_{i,j}$. The contacted individuals are then selected at random from the population.

2.3.5 Simulated overall contact patterns

Figure S5 presents the age-related contact patterns obtained for each contact locations, along with the aggregated contact patterns.

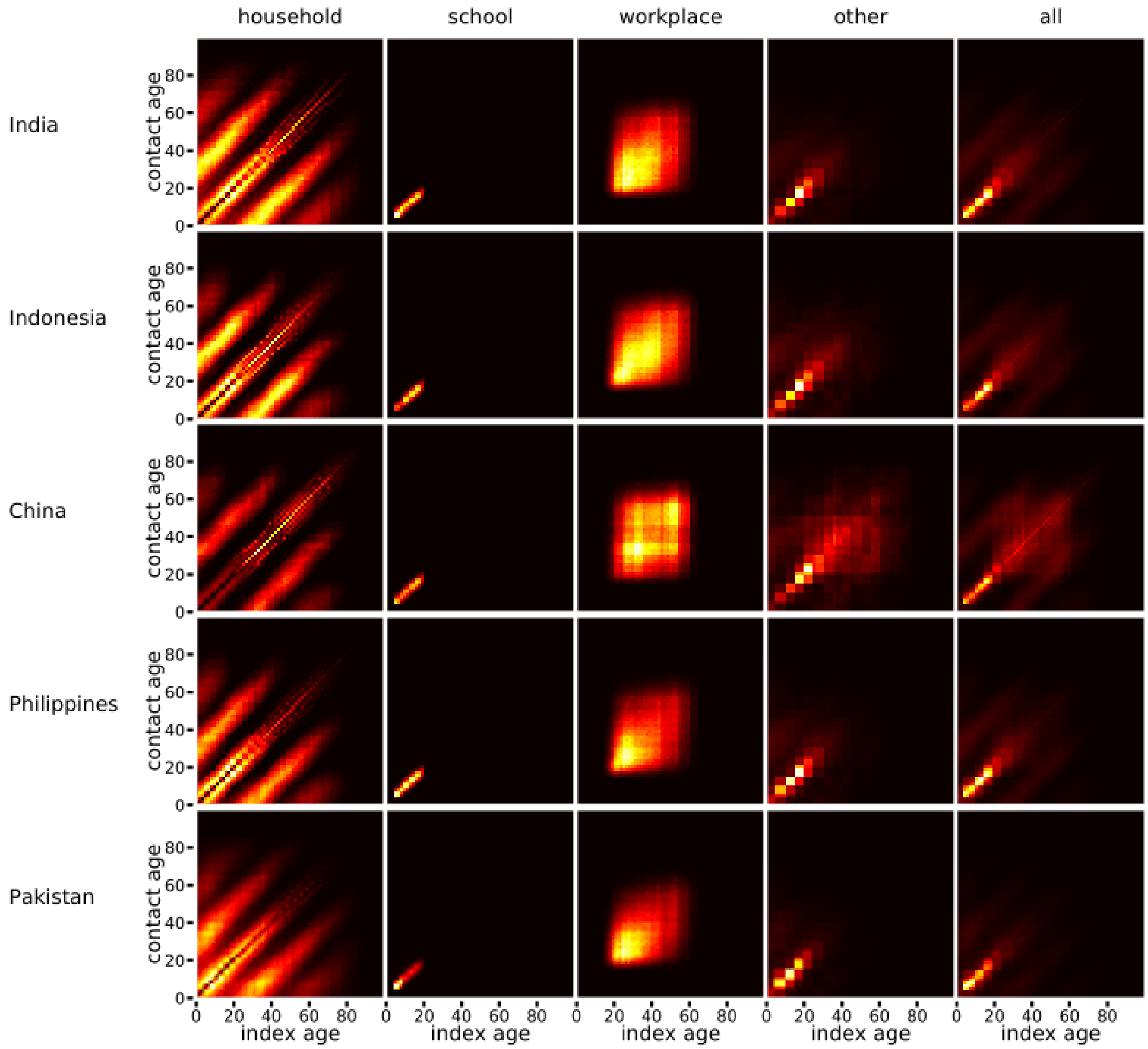

Figure S5. Simulated age-specific contact patterns by country and location.

3 Model of tuberculosis

3.1 Natural history

Once infected with *M. tuberculosis*, individuals may or may not progress to active disease. The estimates obtained from our previous study are used to determine whether and when activation occurs[10]. On development of active TB, an organ-status (smear-positive, smear-negative or extrapulmonary) is randomly assigned to the diseased individual using a multinomial distribution (Table 2, main text). The natural outcome of the disease episode is drawn using rates of TB-specific mortality and self-recovery estimated using data on untreated TB patients' prognosis from the pre-chemotherapy era[15]. A separate study was dedicated to the estimation of the natural history parameters and provided estimates for smear-positive and smear-negative patients[25]. In the present work, we assume that the prognosis of extrapulmonary TB is similar to that of smear-negative TB. Table S4 presents the parameter values estimated in the separate analysis and used to characterise the natural history of TB in our model.

	Smear-positive TB	Smear-negative or extrapulmonary TB
**TB-specific mortality rate (per year)**	0.390 (0.330-0.453)	0.025 (0.016-0.036)
**Self-recovery rate (per year)**	0.234 (0.178-0.294)	0.148 (0.085-0.242)

Table S4. Parameters used to characterise the natural history of TB.
Numbers in brackets represent the 95% confidence intervals.

Exponential distributions (parameterised with the rates above) are used to generate a time to spontaneous recovery and a time to TB-induced death for each individual with TB and we retain the type of disease outcome associated with the shortest duration.

3.2 *M. tuberculosis* transmission

M. tuberculosis transmission may occur when an active TB case contacts a susceptible individual (see Section 2.3 for social contact description). A parameter is used to define the crude probability that a social contact results in effective contact and therefore leads to transmission (Table 1, main text). This parameter is automatically calibrated to reproduce observed national TB prevalence aggregated for all ages as described in Section 4.

The crude transmission probability is then adjusted by contact location (household, school, …) to account for various levels of contact intensities. We first derive the proportions of high-intensity contacts by location using data from a contact survey in Viet Nam and follow the authors' suggestion that the proportions of physical contact reported for each location can be used as a proxy for the proportions of social contacts that are of high-intensity with regard to contact duration and frequency.[22,26] We then assume that low-intensity contacts are half as likely to lead to transmission as high-intensity contacts (Table S1) in the base case. However, two alternate assumptions are also explored in sensitivity analyses regarding this aspect:

- SA 1: Only high-intensity contacts result in transmission.
- SA 2: Low-intensity contacts are assigned the same transmission probability as high-intensity contacts.

The probabilities of transmission per contact may then be adjusted depending on the characteristics of the two individuals involved in the contact. First, active TB cases who are extrapulmonary TB are assumed to be non-infectious (probability of transmission = 0) while a sigmoidal function ($age \longmapsto \frac{1}{1+e^{-(age-a)}}$) is used to model a progressive infectiousness increase with age (Figure S6).[27-29] The parameter a of the sigmoidal function is allowed to vary between 10 and 20 during the calibration process (see Section 4), covering the range of infectiousness profiles presented in Figure S6.

Next, smear-negative TB cases are assumed to be less infectious than smear-positive cases and the associated transmission probability is therefore multiplied by the parameter defining the relative infectiousness of smear-negative cases as compared to smear-positive cases (Table 1, main text). In addition, the transmission probability is assumed to be reduced if the index TB case has been detected (Table 1, main text), reflecting simple preventive measures implemented following TB diagnosis.

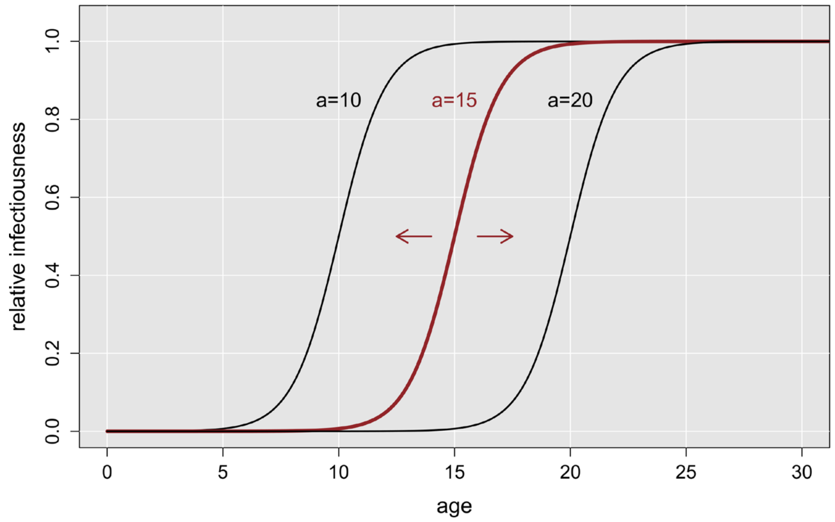

Figure S6. Assumed profiles of infectiousness over age.
The reference used to define relative infectiousness is the infectiousness of adult individuals. The parameter a of the sigmoidal function represents the age at which the relative infectiousness reaches 50%.

The level of susceptibility of the contacted individual may be further reduced if the individual is currently infected with *M. tuberculosis* (Table 1, main text) or has been vaccinated at birth with Bacillus Calmette-Guérin (BCG). We use the profile described in Figure S7 to simulate the wane of BCG efficacy over time from vaccination, based on estimates provided by a retrospective population-based cohort study in Norway,[16] and a controlled clinical trial conducted in England in 1950.[17] Although the latter trial reports on vaccination provided to adolescents, there is evidence to suggest that age at vaccination is not an important predictor of BCG efficacy.[30] Also, note that although Nguipdop-Djomo and colleagues suggest that BCG could possibly be effective for extended periods of time, their estimates of vaccine efficacy were not significant 20-30 years and 30-40 years after vaccination.[16] We therefore make the assumption that BCG is no longer effective 30 years after vaccination.

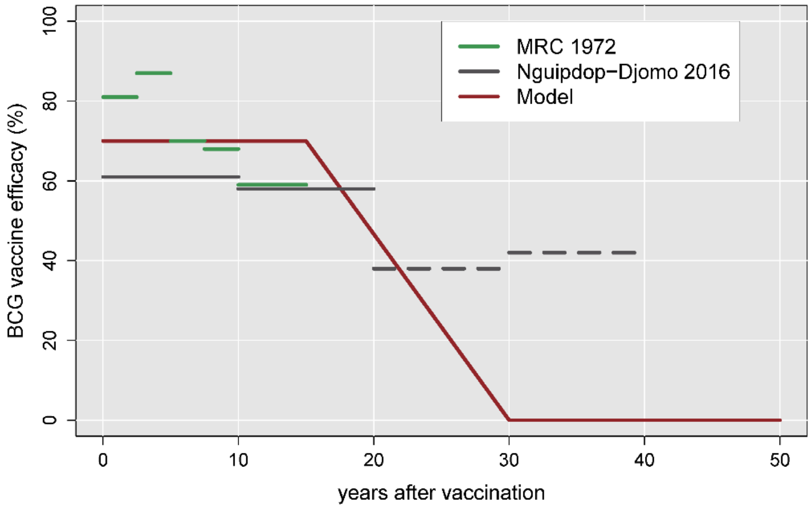

Figure S7. Assumed wane profile of BCG efficacy.
Green and grey lines represent estimates obtained from literature (MRC 1972[17] and Nguipdop-Djomo 2016[16]) while the red line shows the simulated vaccine effect. Dashed lines show literature estimates associated with non-significant efficacy.

3.3 Detection and treatment of active TB

In addition to TB natural history events (described in Section 3.1), events related to TB detection and treatment are simulated for each diseased individual. A detection time is generated for each TB case, although detection only actually occurs if this time precedes the pre-determined time of spontaneous clearance or death. The time from TB disease onset to detection is generated from an exponential distribution. Its parameterisation relies on the value of the programmatic case detection rate, as well as the parameters defining TB natural history. (Sections 3.3.1 and 3.3.2 describe the calculation of this exponential distribution.)

3.3.1 Case detection rate (proportion)

Let λ_N denote the rate of the exponential distribution generating the time to the natural outcome of TB (spontaneous clearance or TB death). Let λ_D denote the rate of the exponentially distributed variable t_D representing the time to TB detection. A TB case will ever become detected if and only if $t_D \leq t_N$. Thus, the case detection rate CDR can be obtained from:

$$
\begin{aligned}
CDR &= P(\, t_D \leq \, t_N) \\
&= P(\exists t \geq 0; \{\, t_D = t\} \cap \{t \leq t_N\}) \\
&= \int_0^{+\infty} f_D(t).(1 - F_N(t)).dt = \int_0^{+\infty} \lambda_D e^{-\lambda_D t}.e^{-\lambda_N t}.dt \\
&= \frac{\lambda_D}{\lambda_D + \lambda_N}
\end{aligned}
$$

3.3.2 Calculation of the rate of the exponential process associated with the time to detection

As the case detection rate (proportion) is more commonly available than the average time to detection, the input parameter used in the model is the CDR. That is, the rate of the exponential distribution used to generate the time to detection is obtained by rearranging the latest equation to obtain:

$$\lambda_D = \frac{CDR}{1 - CDR} \lambda_N.$$

Note that the detection parameter λ_D differs according to the type of disease, as TB natural history (characterised by λ_N) is different for smear-positive cases compared to smear-negative or extrapulmonary cases. Specifically, assuming the same CDR for all forms of TB leads to a greater average time to detection of smear-negative and extrapulmonary cases.

3.3.3 Treatment of active TB

Detected cases of TB are assumed to be started on treatment between zero and 14 days after detection. In case of successful completion of treatment, infection is assumed to be cleared as a consequence of treatment, such that the individual returns to a susceptible state. If treatment fails, the patient's TB will remain active and the individual's outcome will be defined by his/her associated TB natural history as initially generated (Section 3.1).

3.4 Time-variant programmatic parameters

In order to account for the changing characteristics of TB control in the different countries, we allow parameter values to vary over time for three important programmatic indicators: BCG vaccination coverage, case detection rate and treatment success rate. Data extracted from the WHO (case detection and treatment success rates) and

UNICEF (vaccination) databases are used to fit the associated scale-up curves (Figure S8). These time-variant parameters are used during the TB burn-in phase, simulating the period 1918-2018.

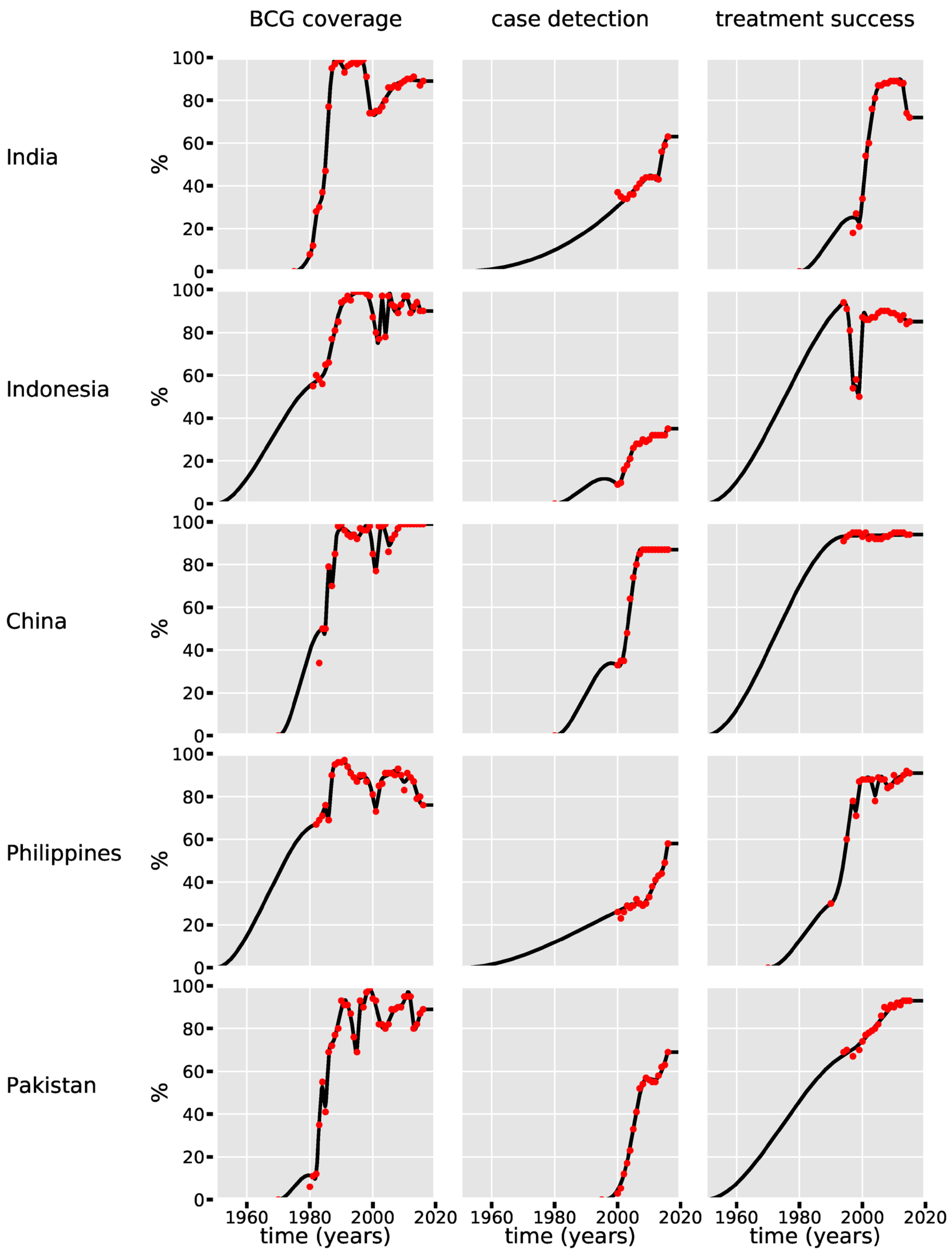

Figure S8. Fitting of time-variant programmatic parameters to data.
Red dots: data. Black line: scale-up function

4 Model calibration

4.1 Description

Due to the stochastic nature of the model, a single set of parameters would inevitably lead to different outcomes for different runs, generating uncertainty in the model outputs. In addition to the uncertainty arising from stochasticity, we accounted for the uncertainty surrounding several parameter values. We quantified the uncertainty around 11 important epidemiological parameters (Table S5) that were varied during model calibration. The 11 parameters were selected in order to capture the main stages that characterise the natural history of *M.tb* transmission, infection progression and TB disease, and to allow for uncertainty in the processes likely to affect the epidemic age-profile of TB (age-dependent infectiousness and differential risk of progression towards active TB). We also included parameters that were not informed by strong evidence and therefore associated with important uncertainty.

Parameter	Details	Prior distribution	Source
Probability of transmission per contact	For contact between a diseased individual and a susceptible one. Before adjustment for differential infectiousness or susceptibility (see Section 3.2).	Uniform on [0.002, 0.008]	Range determined from preliminary calibrations.
Parameter a characterising the age-profile of infectiousness	See detailed description in Section 3.2 and Figure S6. a represents the age at which infectiousness is assumed to reach half that of adult individuals.	Triangular on [10, 20], mode=15	[27-29]
Proportion of infected individuals aged 0-4 at low risk of progression to active TB	Using the conceptualisation of TB latency associated with Model 6 in Ragonnet et al.[10]	Beta(73, 40)	[10]
Proportion of infected individuals aged 5-14 at low risk of progression to active TB	Idem	Beta(147, 35)	[10]
Proportion of infected individuals aged 15 and older at low risk of progression to active TB	Idem	Beta(584, 29)	[10]
Rate of TB-specific mortality for smear-positive TB (per year)	See section 3.1	Triangular on [0.31, 0.47], mode=0.39*	[15,25]
Rate of TB-specific mortality for smear-negative and extrapulmonary TB (per year)	See section 3.1	Triangular on [0.013, 0.039], mode=0.025*	[15,25]
Rate of self-recovery for smear-positive TB (per year)	See section 3.1	Triangular on [0.16, 0.31], mode=0.23*	[15,25]
Rate of self-recovery for smear-negative and extrapulmonary TB (per year)	See section 3.1	Triangular on [0.05, 0.25], mode=0.15*	[15,25]
Time from detection to treatment (days)		Triangular on [0, 14], mode=7	[31-34]
Average number of potential contacts at work		Triangular on [10, 30], mode=20	Assumption

Table S5. Uncertainty parameters and their prior distributions.
*The statistical distributions were determined to match the 95% confidence intervals presented in Table S4.

For each country, we used Latin Hypercube Sampling (LHS) to draw 100 distinct parameter sets, using the prior distributions presented in Table S5. Next, we ran the model numerous times for each of the sampled parameter sets and we retained only the simulations that satisfied the calibration conditions described in Section 4.2. The number of simulations per parameter set was not determined a priori. Instead, we repeated simulations until we obtained 200 calibrated models (in total) for each country, which generally required running around 40 simulations per parameter set (i.e. 4,000 simulations overall per country). The results presented in the manuscript are issued from the 200 calibrated simulations per country.

4.2 Calibration targets

We used the TB prevalence estimates obtained from national TB prevalence surveys in Indonesia (years 2004 and 2014), China (2000 and 2010), the Philippines (2007 and 2016) and Pakistan (2011). For India, as no national prevalence survey has been conducted prior to the one that is currently ongoing and yet to report, we used the TB prevalence estimates reported in the latest national TB report. Table S6 summarises the country-specific estimates used for calibration.

Country	Year	TB prevalence (/100,000 population)	Comment	Source
India	2015	320 (280-380)		India TB Report 2018[35]
Indonesia	2004	790 (626-973)		NTPS*[36] and WHO[37]
	2014	647 (513-797)		NTPS[36] and WHO[37]
China	2000	170 (146-196)		NTPS[38] and WHO[37]
	2010	108 (94-123)		NTPS[38] and WHO[37]
Philippines#	2007	260 (170-360)	Only smear-positive TB	NTPS[39]
	2016	434 (350-518)	Only smear-positive TB	NTPS[40]
Pakistan	2011	348 (291-410)		NTPS[41] and WHO[37]

Table S6. Estimates of TB prevalence used for calibration.
95% confidence intervals are presented in brackets. *NTPS: national tuberculosis prevalence survey. #Estimates of smear-positive TB only were used in the Philippines due to the important changes of smear-negative case definition between 2007 and 2016.

During the calibration process described in Section 4.1, we retained the simulations that produced prevalence estimates falling into the ranges presented in Table S6 for the relevant year(s).

4.3 Posterior parameter ranges

Figure S9 represents the median value and 95% simulation intervals associated with the 11 parameters varied during calibration.

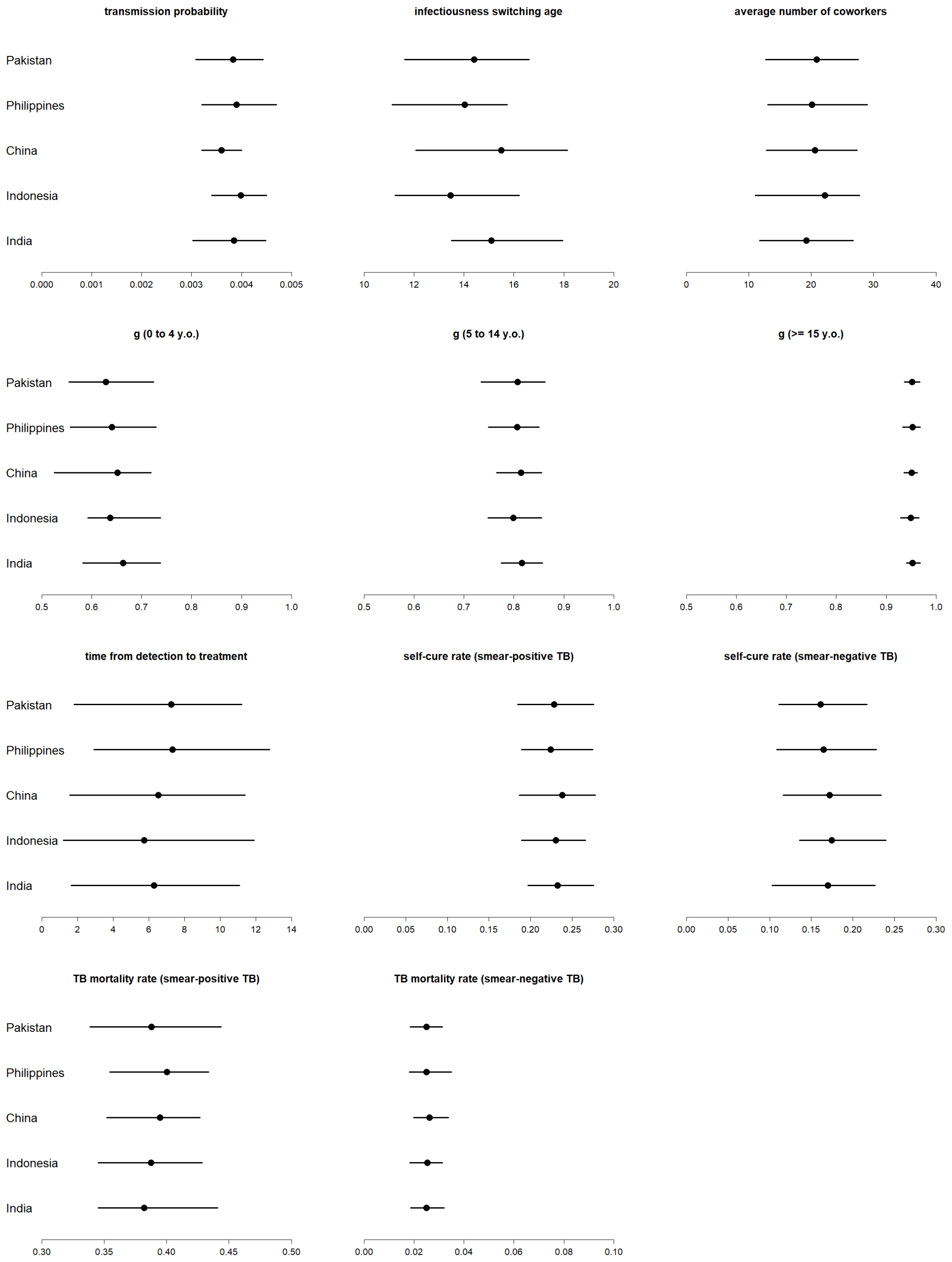

Figure S9. Posterior parameter ranges associated with the 100 calibrated simulations per country.

5 Additional results

5.1 Estimated age-specific active TB prevalence for India

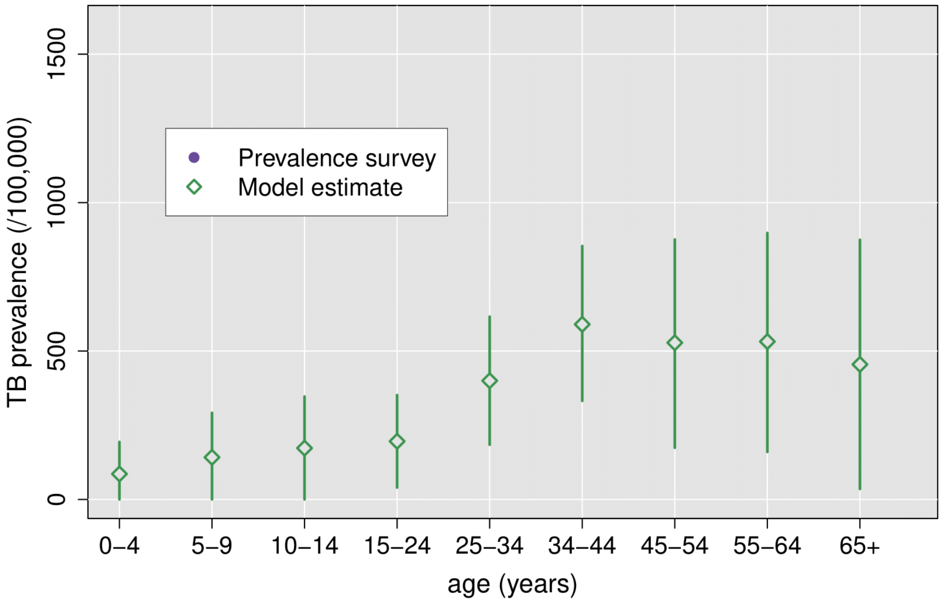

Figure S10. Age-specific active TB prevalence for India in 2015
Error bars represent the 95% simulation intervals

5.2 Estimated prevalence of latent TB infection

Although estimating prevalence of latent TB infection (LTBI) was not among the objectives of the study, we conducted a comparison of our model outputs with previously published estimates for country-level prevalence of LTBI for independent validation purpose.

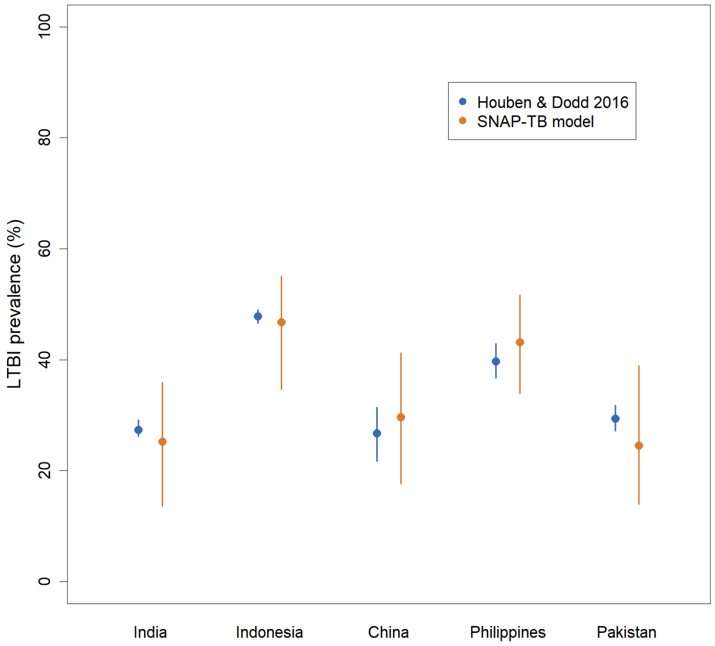

Figure S11. Estimated LTBI prevalence according to our model versus *Houben & Dodd 2016*.
The vertical bars represent the 95% confidence intervals.

5.3 Considering an alternative age-specific infectiousness profile

This section presents the results of our analysis performed considering a different age-specific profile of infectiousness as presented on Figure S12.

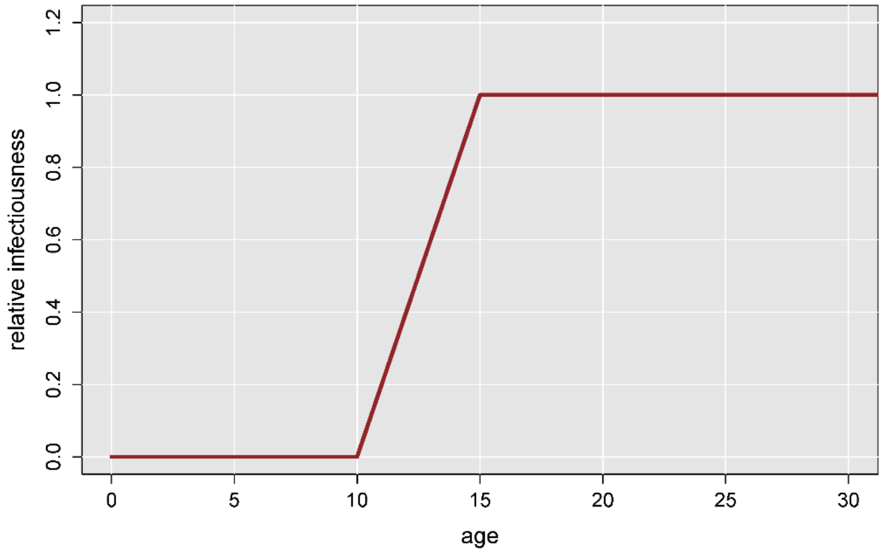

Figure S12. Alternative age-specific infectiousness profile for sensitivity analysis

This profile is based on a linear infectiousness increase between the ages of 10 and 15 years old. The age-category 10-15 years old is now assumed to be more infectious compared to the baseline assumption presented in Figure S6.

Figure S13 to Figure S16 present the results associated with this sensitivity analysis for the five countries.

This sensitivity analysis shows similar findings to those obtained at baseline. However, we note a higher TB burden in the 10-15 year-old population under this scenario, which highlights the sensitivity of our estimates regarding the young populations to the assumed infectiousness profile. The other model outputs pertaining to transmission and LTBI age-distribution were unaffected by the change in infectiousness profile.

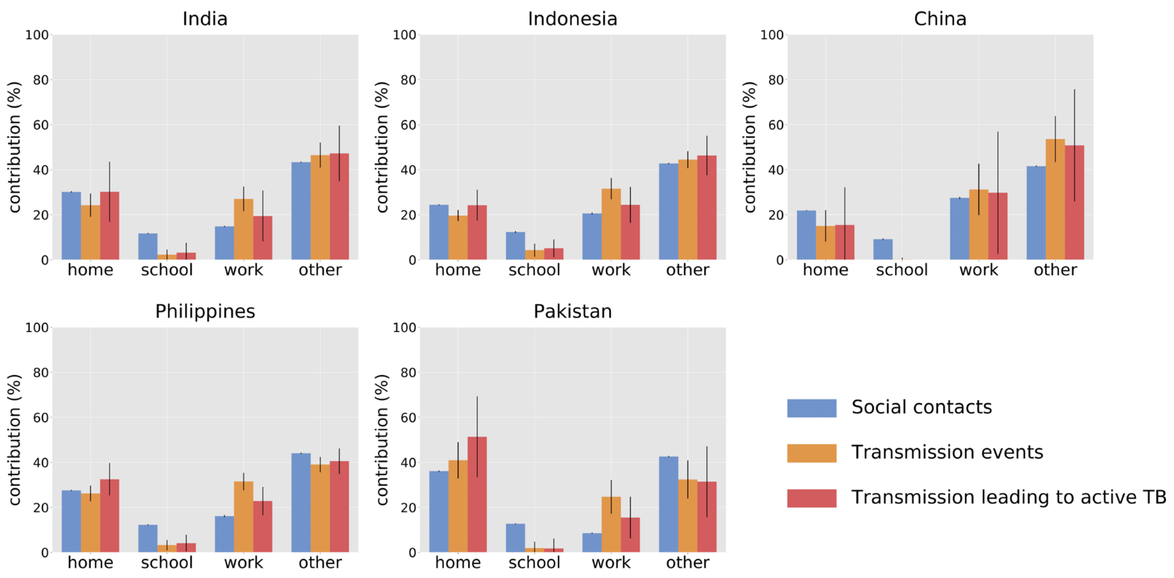

Figure S13. Contributions of the various locations to the burden of contact and transmission (alternative infectiousness profile)
Error bars represent the 95% confidence intervals obtained from simulation.

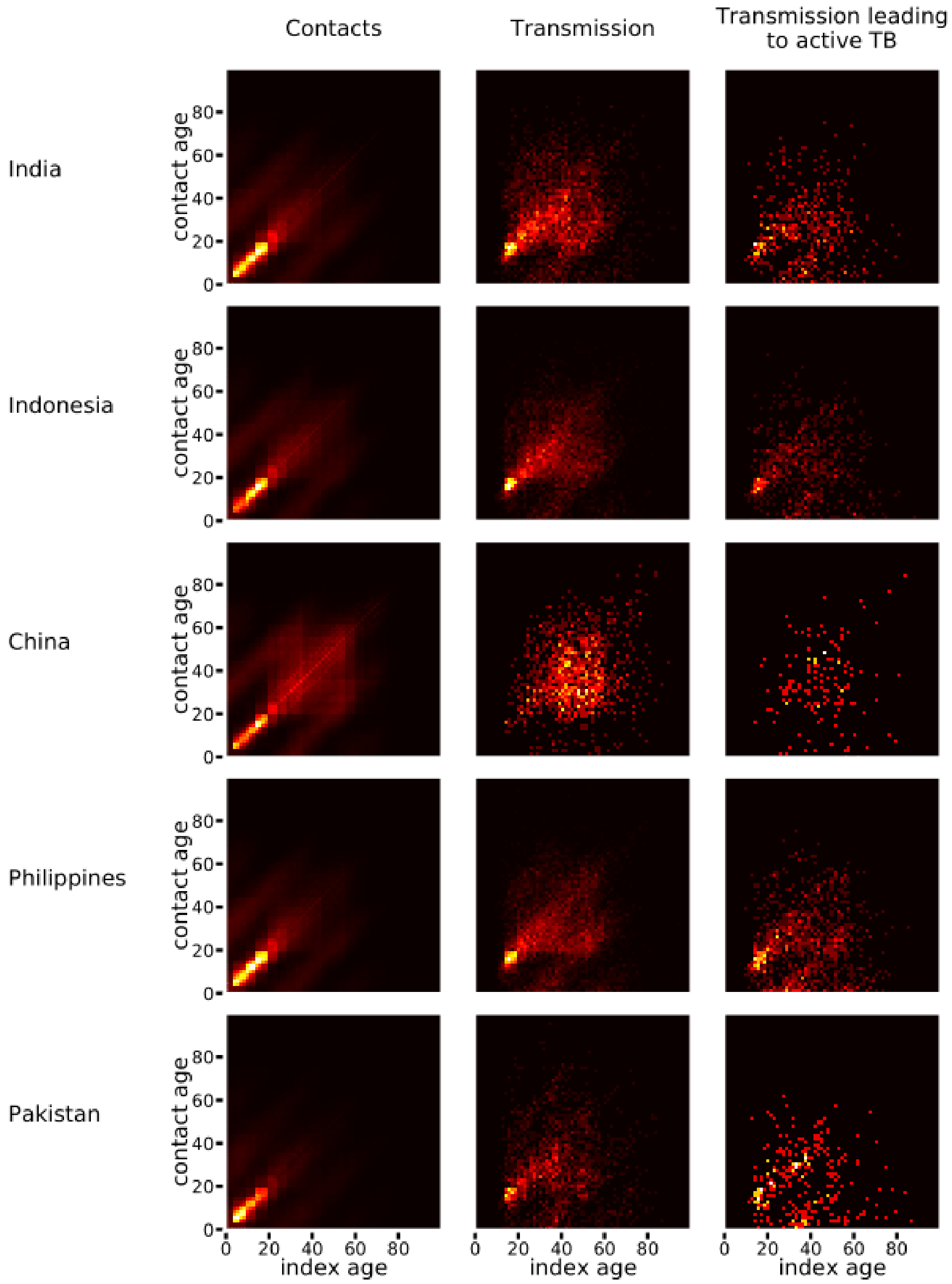

Figure S14. Age-specific profile of social mixing and transmission (alternative infectiousness profile)

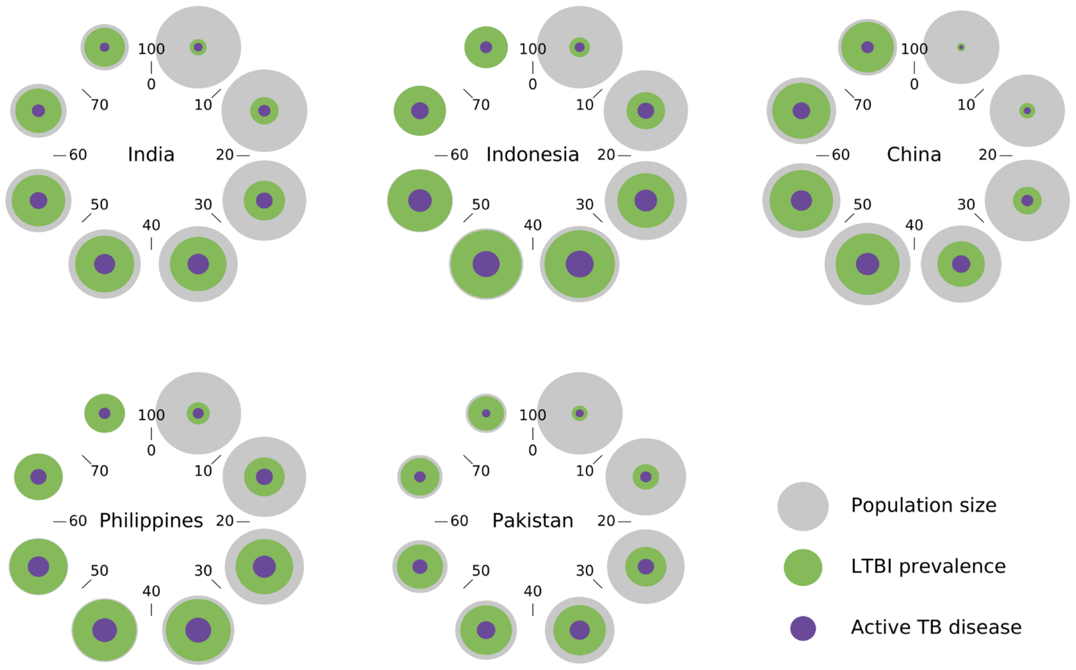

Figure S15. Age-distribution of latent tuberculosis infection (alternative infectiousness profile)
The volume of the spheres is proportional to the following quantities: 2018 population (grey), latent tuberculosis infection (LTBI) prevalence in 2018 (green), and number of individuals currently infected in 2018 who will ever develop active TB (purple).

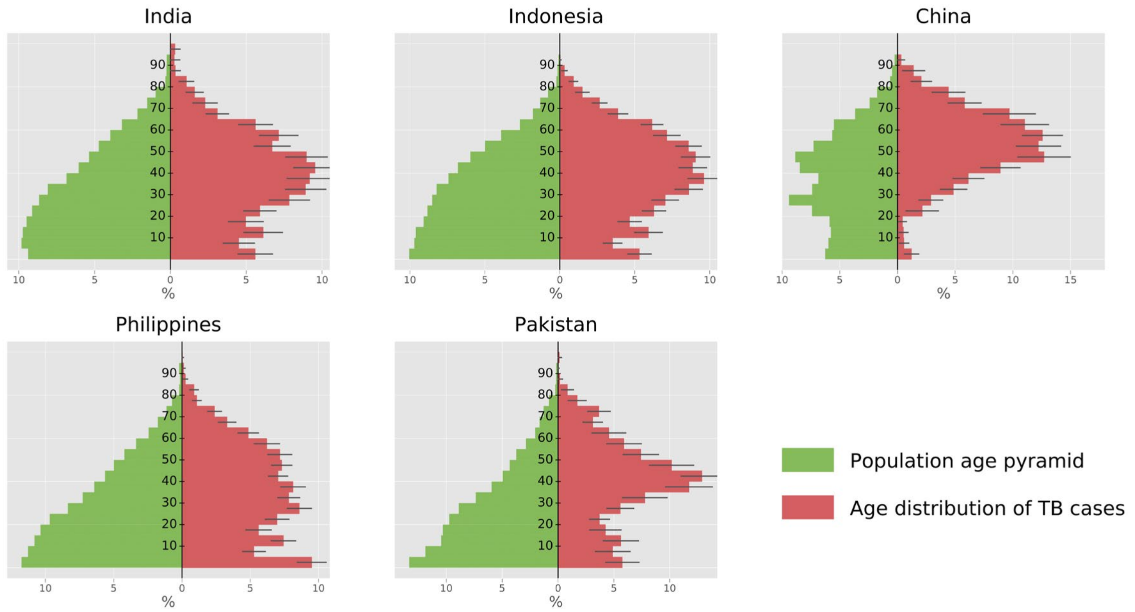

Figure S16. Age-distribution of TB cases (alternative infectiousness profile)
Age of TB cases at activation (red) were recorded over a period of five years starting from 2018. Error bars represent the 95% confidence intervals obtained from simulation for the TB age-distribution.

5.4 Ignoring the past programmatic background

The results presented in this section relate to our analysis performed without time-variant parameters such that the programmatic background is supposed to have remained unchanged over time and equal to the most recent one. This analysis was applied to the Philippines.

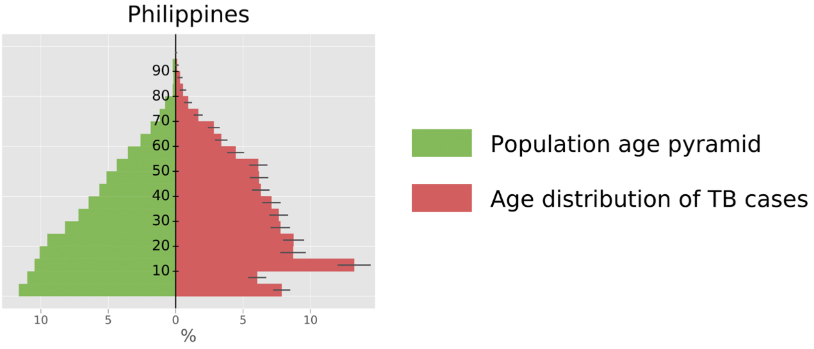

Figure S17. Results for the Philippines when no time-variant parameters are included.

5.5 Alternative scenarios for the risk of transmission through low- versus high-intensity contacts

This section presents the results of the two sensitivity analyses that consider alternative relative risks of transmission through low-intensity contacts as compared to high-intensity contacts:

- SA 1: Only high-intensity contacts result in transmission.
- SA 2: Low-intensity contacts are assigned the same transmission probability as high-intensity contacts.

5.5.1 Only high-intensity contacts can result in transmission (SA 1)

In this sensitivity analysis, we assume that low-intensity contacts cannot lead to *M. tb* transmission.

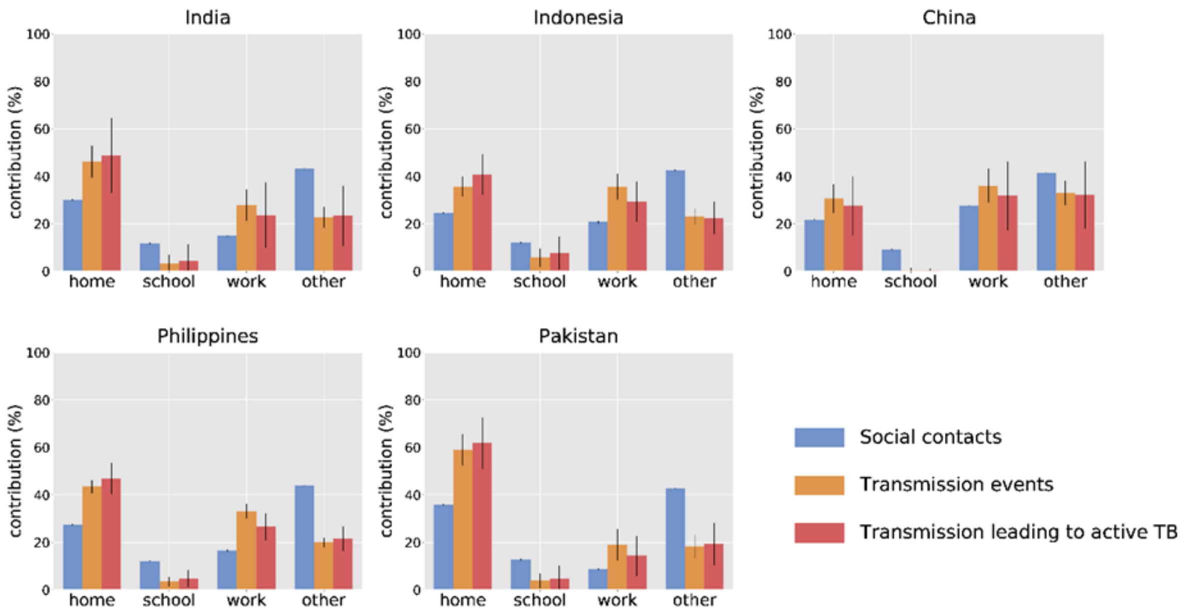

Figure S18. Contributions of the various locations to the burden of contact and transmission (scenario SA 1)

Error bars represent the 95% confidence intervals obtained from simulation.

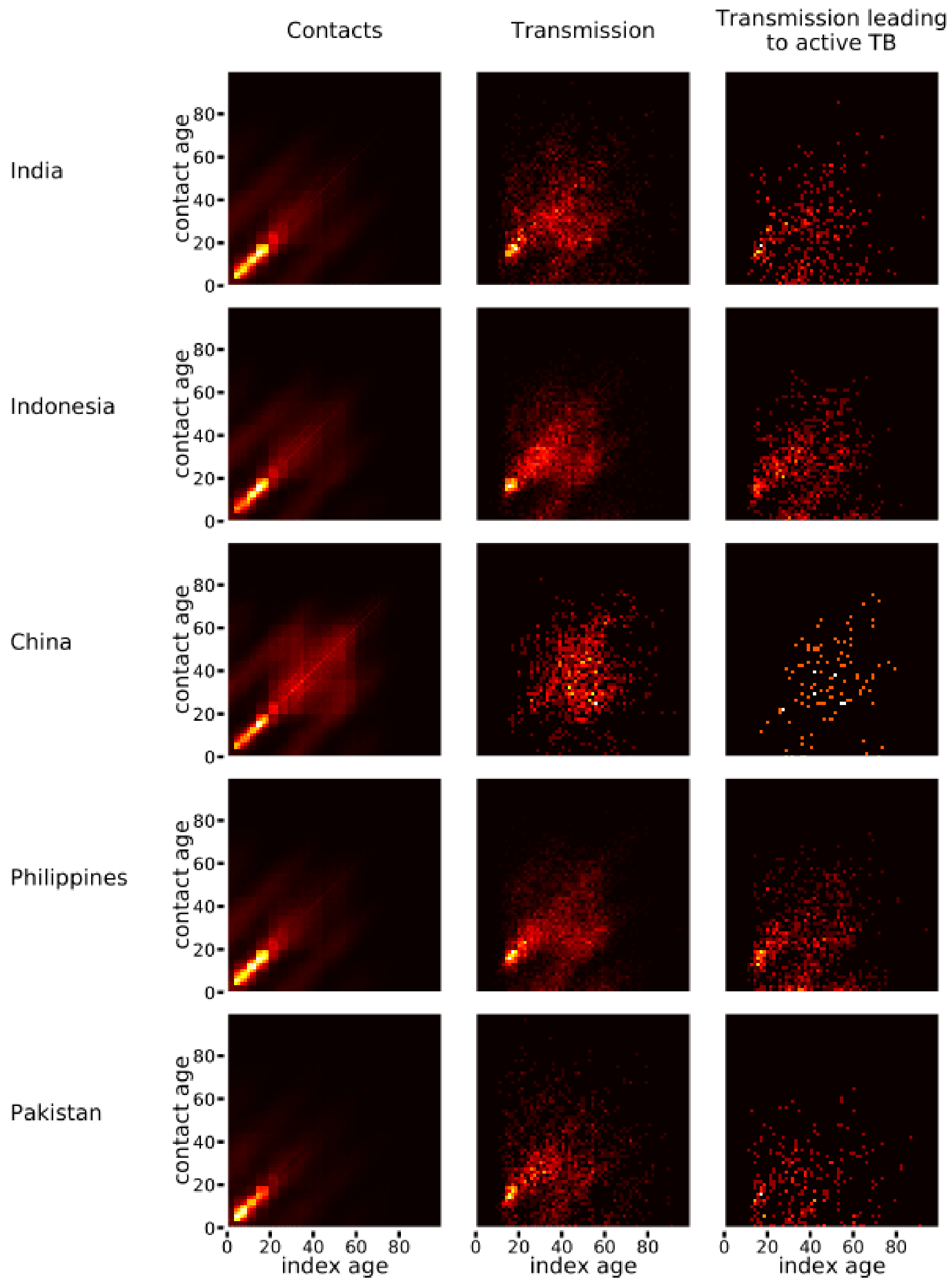

Figure S19. Age-specific profile of social mixing and transmission (scenario SA 1)

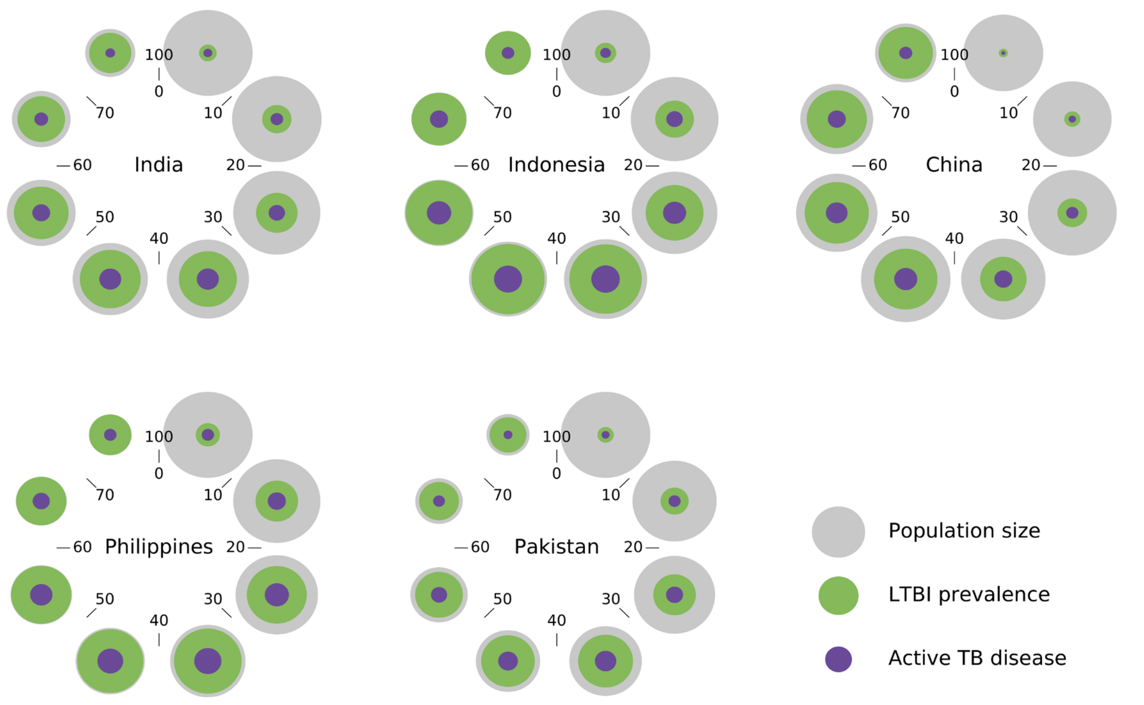

Figure S20. Age-distribution of latent tuberculosis infection (scenario SA 1)
The volume of the spheres is proportional to the following quantities: 2018 population (grey), latent tuberculosis infection (LTBI) prevalence in 2018 (green), and number of individuals currently infected in 2018 who will ever develop active TB (purple).

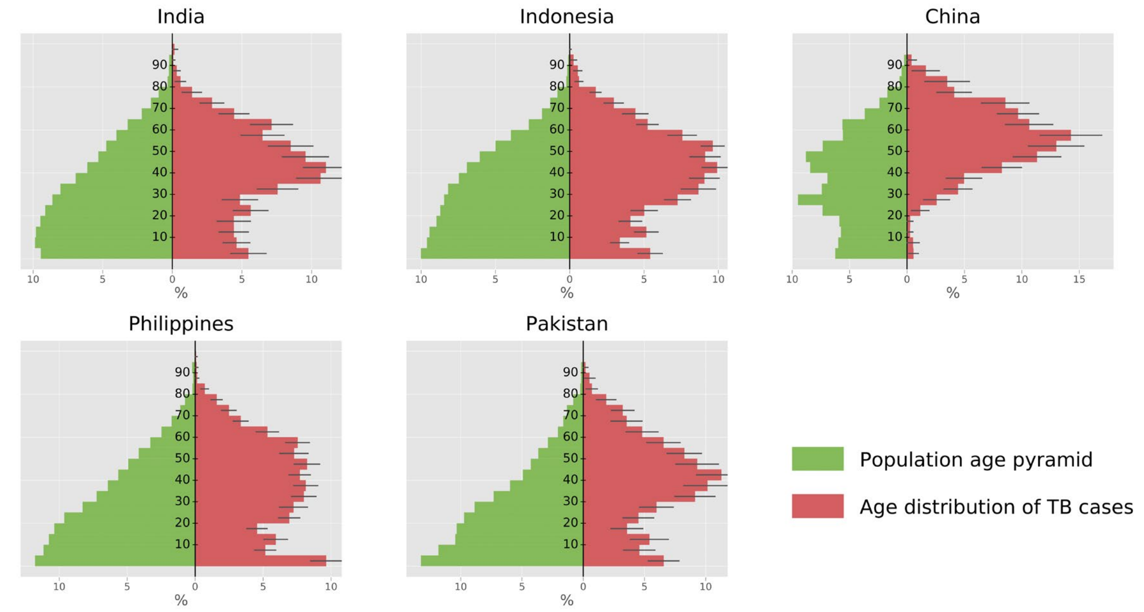

Figure S21. Age-distribution of TB cases (scenario SA 1)
Age of TB cases at activation (red) were recorded over a period of five years starting from 2018. Error bars represent the 95% confidence intervals obtained from simulation for the TB age-distribution.

5.5.2 Equal risk of transmission for low- and high-intensity contacts (SA 2)

In this sensitivity analysis, we assume that the risk of transmission for low-intensity contacts is equal to that of high-intensity contacts.

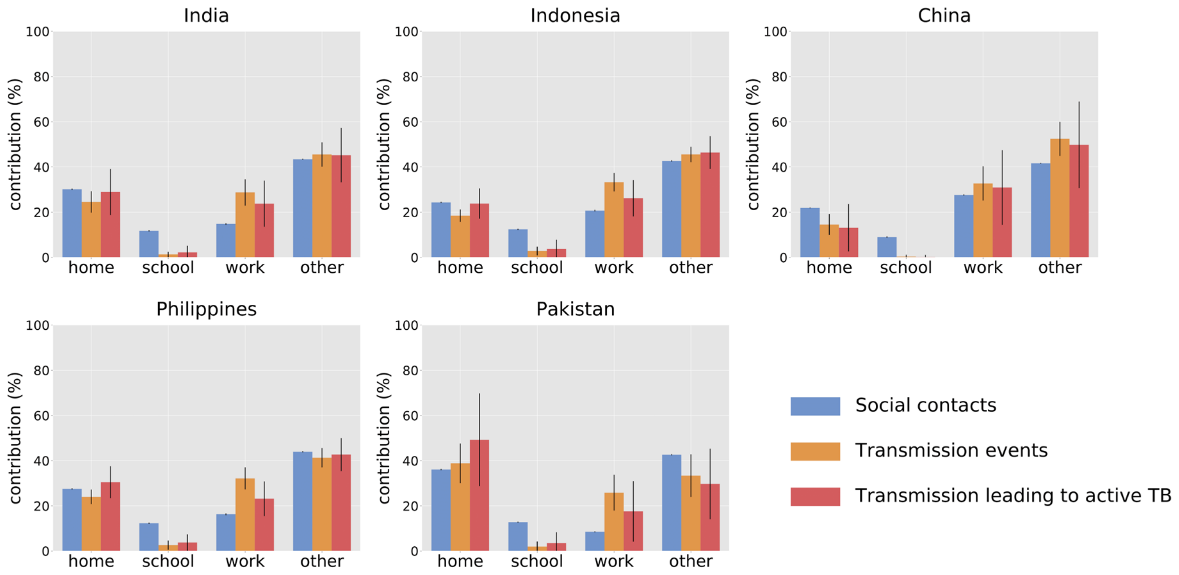

Figure S22. Contributions of the various locations to the burden of contact and transmission (scenario SA 2)

Error bars represent the 95% confidence intervals obtained from simulation.

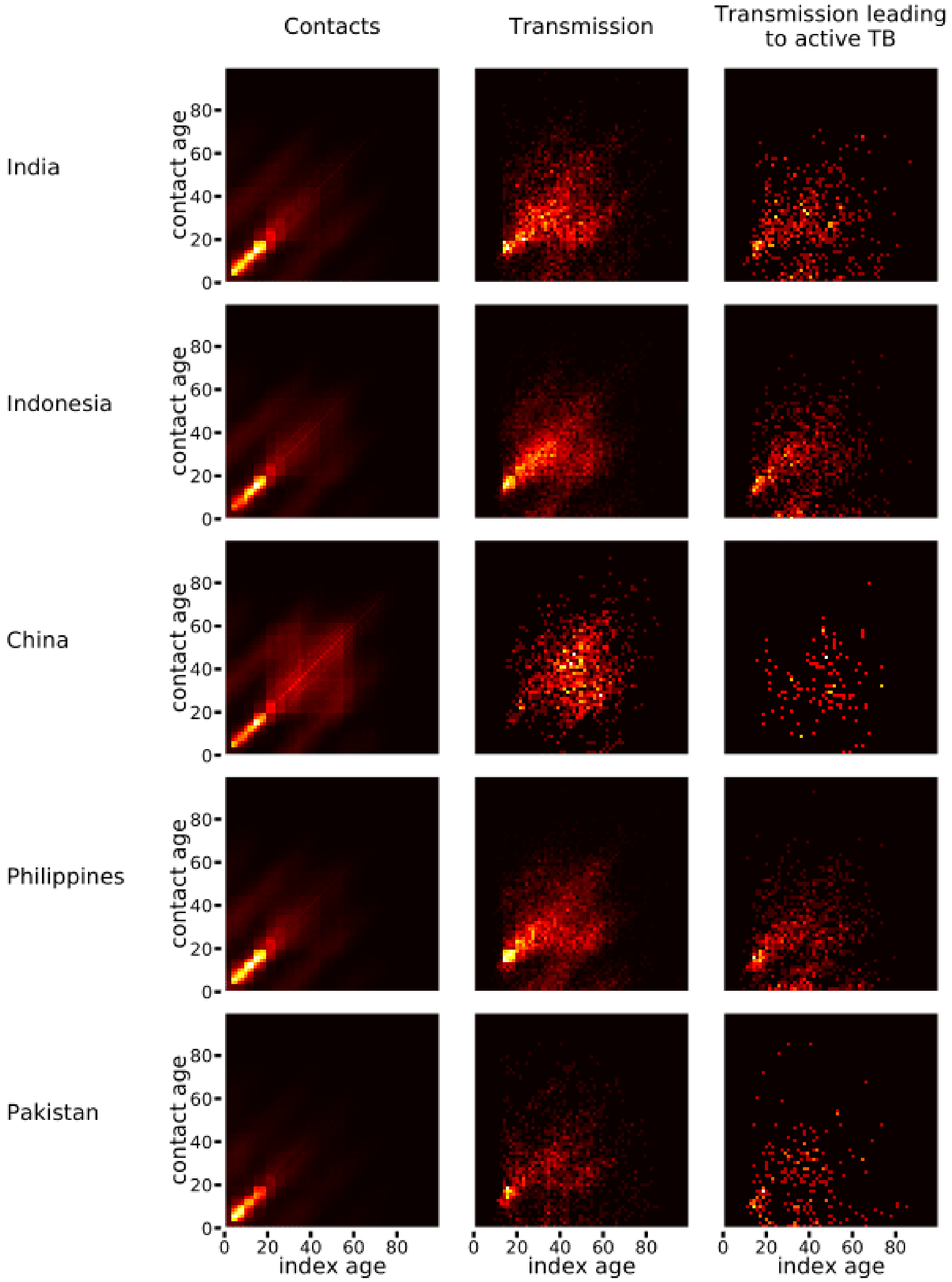

Figure S23. Age-specific profile of social mixing and transmission (scenario SA 2)

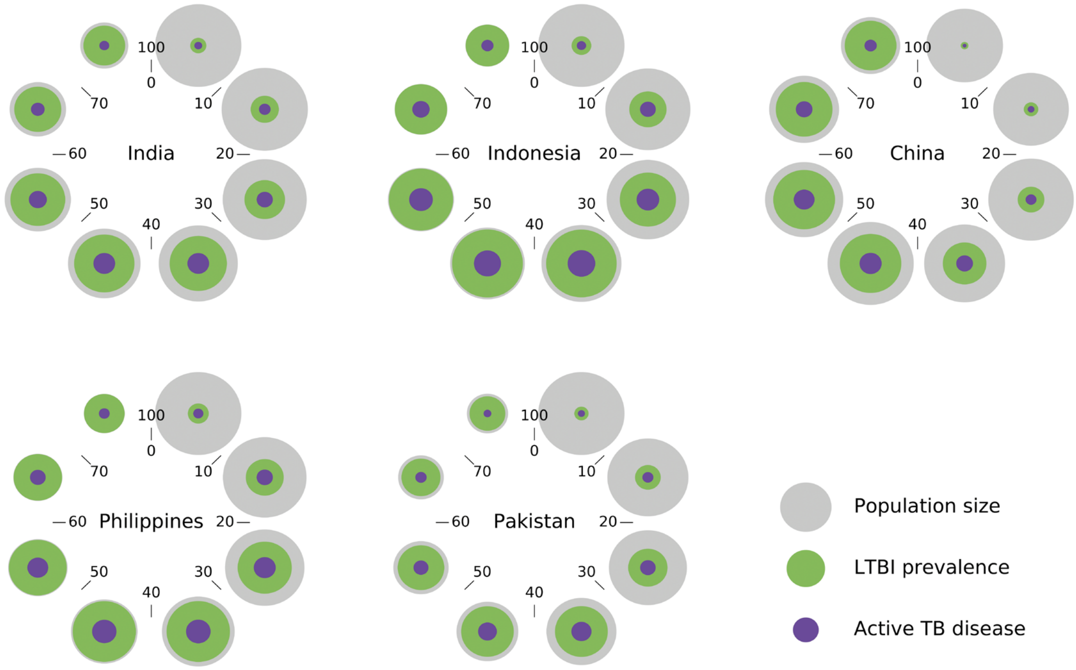

Figure S24. Age-distribution of latent tuberculosis infection (scenario SA 2)
The volume of the spheres is proportional to the following quantities: 2018 population (grey), latent tuberculosis infection (LTBI) prevalence in 2018 (green), and number of individuals currently infected in 2018 who will ever develop active TB (purple).

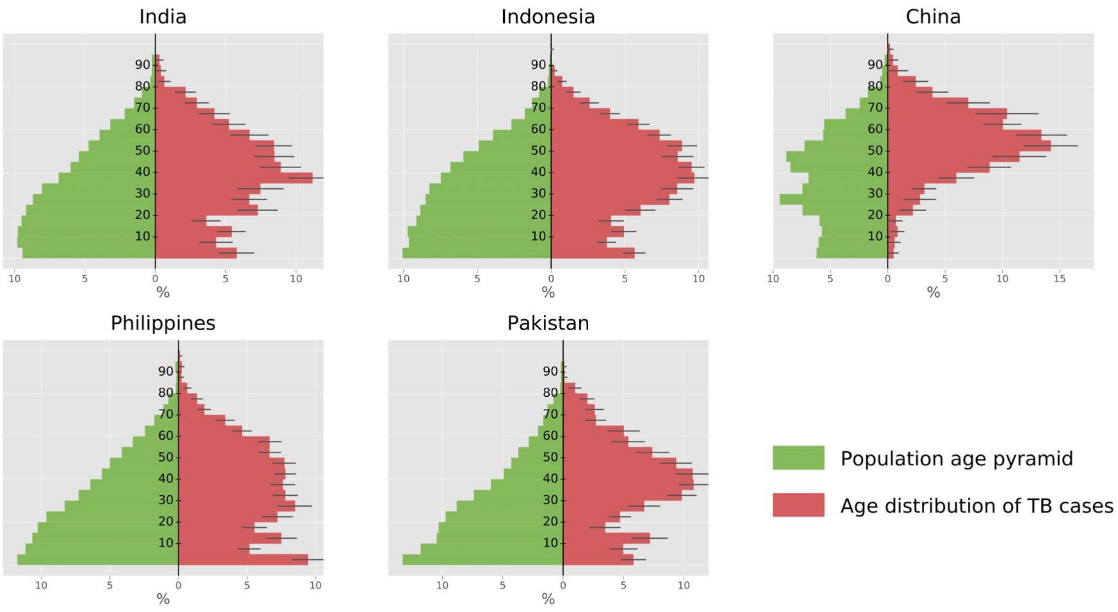

Figure S25. Age-distribution of TB cases (scenario SA 2)
Age of TB cases at activation (red) were recorded over a period of five years starting from 2018. Error bars represent the 95% confidence intervals obtained from simulation for the TB age-distribution.

5.5.3 Findings from sensitivity analyses SA 1 and SA 2

When assuming that only high-intensity contacts can result in transmission, household contacts become the predominant driver of *M.tb* transmission in all countries except China (Figure S18). We also observe an

increased TB burden affecting the 0-5 year-old category as a result of the enhanced contribution of household transmission. In contrast, assuming that the risk of transmission is independent of contact intensity results in a greater contribution of "other locations" to the TB burden as compared to the baseline scenario. As a consequence, the relative TB burden in those aged 40 years and above is diminished due to the lower frequency of contacts in "other locations" for this age-group.

Figure S26 presents the calibrated crude probability of transmission per contact for the different scenarios (baseline, SA 1, SA 2). We note that the value of this parameter is found to be very similar in each of the five countries regardless of the scenario considered. However, as anticipated, higher crude transmission probabilities are required to reach the calibration targets when assuming that only high-intensity contacts can result in transmission (SA 1). In contrast, the scenario SA 2 assuming that low-intensity contacts have the same transmission probability as high-intensity contacts is associated with lower crude transmission probabilities.

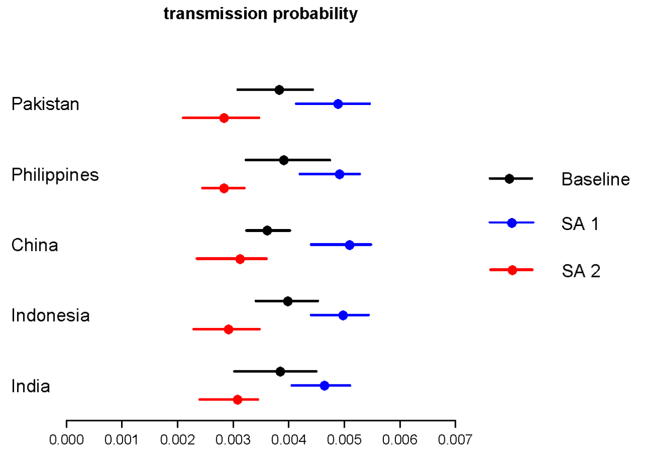

Figure S26. Calibrated probabilities of transmission per contact for sensitivity analyses.
The dots and bars represent the median estimates and 95% simulation intervals. The baseline scenario is represented in black, the sensitivity analysis SA 1 in blue and the sensitivity analysis SA 2 in red.

6 References

1. Prem K, Cook AR, Jit M. Projecting social contact matrices in 152 countries using contact surveys and demographic data. *PLoS Comput Biol* 2017; **13**(9): e1005697.

2. The United Nations - Population Division. Household size and composition. 2017.

3. Republic of The Philippines Department of Education. Number of schools. 2018. http://www.deped.gov.ph/.

4. Shaweno D, Karmakar M, Alene KA, et al. Methods used in the spatial analysis of tuberculosis epidemiology: a systematic review. *BMC Med* 2018; **16**(1): 193.

5. China Ministry of Education. Number of schools. 2018. http://en.moe.gov.cn/Resources/Statistics/edu_stat_2016/2016_en01/201708/t20170823_311669.html.

6. Pakistan Education Statistics. Number of schools. 2016.

7. The World Bank - Labor force participation rate. 2018.

8. The World Bank. World Bank and Education in Indonesia. 2014. http://www.worldbank.org/en/country/indonesia/brief/world-bank-and-education-in-indonesia.

9. Sloot R, Schim van der Loeff MF, Kouw PM, Borgdorff MW. Risk of Tuberculosis after Recent Exposure. A 10-Year Follow-up Study of Contacts in Amsterdam. *Am J Respir Crit Care Med* 2014; **190**(9): 1044-52.

10. Ragonnet R, Trauer JM, Scott N, Meehan MT, Denholm JT, McBryde ES. Optimally capturing latency dynamics in models of tuberculosis transmission. *Epidemics* 2017; **21**: 39-47.

11. Trauer JM, Moyo N, Tay EL, et al. Risk of Active Tuberculosis in the Five Years Following Infection . . . 15%? *Chest* 2016; **149**(2): 516-25.

12. Tostmann A, Kik SV, Kalisvaart NA, et al. Tuberculosis transmission by patients with smear-negative pulmonary tuberculosis in a large cohort in the Netherlands. *Clin Infect Dis* 2008; **47**(9): 1135-42.

13. Behr MA, Warren SA, Salamon H, et al. Transmission of Mycobacterium tuberculosis from patients smear-negative for acid-fast bacilli. *Lancet* 1999; **353**(9151): 444-9.

14. Melsew YA, Doan TN, Gambhir M, Cheng AC, McBryde E, Trauer JM. Risk factors for infectiousness of patients with tuberculosis: a systematic review and meta-analysis. *Epidemiology and infection* 2018: 1-9.

15. Tiemersma EW, van der Werf MJ, Borgdorff MW, Williams BG, Nagelkerke NJ. Natural history of tuberculosis: duration and fatality of untreated pulmonary tuberculosis in HIV negative patients: a systematic review. *PLoS One* 2011; **6**(4): e17601.

16. Nguipdop-Djomo P, Heldal E, Rodrigues LC, Abubakar I, Mangtani P. Duration of BCG protection against tuberculosis and change in effectiveness with time since vaccination in Norway: a retrospective population-based cohort study. *The Lancet infectious diseases* 2016; **16**(2): 219-26.

17. Medical Research Council. BCG and vole bacillus vaccines in the prevention of tuberculosis in adolescence and early adult life. *Bull World Health Organ* 1972; **46**(3): 371-85.

18. WHO. Official Country Estimates of Immunization Coverage for the Year 2016. 2018.

19. WHO. Tuberculosis country profiles. 2017. http://www.who.int/tb/country/data/profiles/en/ (accessed 24/04/2017 2015).

20. WHO. Global tuberculosis report 2017. Geneva: World Health Organization, 2017.

21. Observatory GH. Life tables by country. 2018. http://apps.who.int/gho/data/node.main.LIFECOUNTRY?lang=en (accessed 18/01/2018.

22. Mossong J, Hens N, Jit M, et al. Social contacts and mixing patterns relevant to the spread of infectious diseases. *PLoS Med* 2008; **5**(3): e74.

23. Grijalva CG, Goeyvaerts N, Verastegui H, et al. A household-based study of contact networks relevant for the spread of infectious diseases in the highlands of Peru. *PLoS One* 2015; **10**(3): e0118457.

24. Goeyvaerts N SE, Potter G, Torneri A, Van Kerckhove K, Willem L, Aerts M, Beutels P, Hens N. Household Members Do Not Contact Each Other at Random: Implications for Infectious Disease Modelling. Pre-print.

25. Ragonnet R, Flegg JA, Brilleman SL, et al. Revisiting the Natural History of Pulmonary Tuberculosis: a Bayesian Estimation of Natural Recovery and Mortality rates. *bioRxiv* 2019.

26. Horby P, Pham QT, Hens N, et al. Social contact patterns in Vietnam and implications for the control of infectious diseases. *PLoS One* 2011; **6**(2): e16965.

27. Middelkoop K, Bekker LG, Morrow C, Zwane E, Wood R. Childhood tuberculosis infection and disease: a spatial and temporal transmission analysis in a South African township. *S Afr Med J* 2009; **99**(10): 738-43.

28. Marais BJ, Gie RP, Schaaf HS, Beyers N, Donald PR, Starke JR. Childhood pulmonary tuberculosis: old wisdom and new challenges. *Am J Respir Crit Care Med* 2006; **173**(10): 1078-90.

29. Donald PR. Childhood tuberculosis: the hidden epidemic. *Int J Tuberc Lung Dis* 2004; **8**(5): 627-9.

30. Colditz GA, Brewer TF, Berkey CS, et al. Efficacy of BCG vaccine in the prevention of tuberculosis. Meta-analysis of the published literature. *JAMA* 1994; **271**(9): 698-702.

31. Belkina TV, Khojiev DS, Tillyashaykhov MN, et al. Delay in the diagnosis and treatment of pulmonary tuberculosis in Uzbekistan: a cross-sectional study. *BMC Infect Dis* 2014; **14**: 624.

32. Sreeramareddy CT, Qin ZZ, Satyanarayana S, Subbaraman R, Pai M. Delays in diagnosis and treatment of pulmonary tuberculosis in India: a systematic review. *Int J Tuberc Lung Dis* 2014; **18**(3): 255-66.

33. Asefa A, Teshome W. Total delay in treatment among smear positive pulmonary tuberculosis patients in five primary health centers, southern Ethiopia: a cross sectional study. *PLoS One* 2014; **9**(7): e102884.

34. Jurcev-Savicevic A, Mulic R, Kozul K, et al. Health system delay in pulmonary tuberculosis treatment in a country with an intermediate burden of tuberculosis: a cross-sectional study. *BMC Public Health* 2013; **13**: 250.

35. Division ICT. India TB report 2018, 2018.

36. Ministry of Health RoI. Indonesia tuberculosis prevalence survey 2013-2014, 2015.

37. WHO. Global Tuberculosis Report 2015: World Health Organization, 2015.

38. Wang L, Zhang H, Ruan Y, et al. Tuberculosis prevalence in China, 1990-2010; a longitudinal analysis of national survey data. *Lancet* 2014; **383**(9934): 2057-64.

39. Tupasi TE, Radhakrishna S, Chua JA, et al. Significant decline in the tuberculosis burden in the Philippines ten years after initiating DOTS. *Int J Tuberc Lung Dis* 2009; **13**(10): 1224-30.

40. Health PDo. National tuberculosis prevalence survey 2016 Philippines, 2018.

41. Qadeer E, Fatima R, Yaqoob A, et al. Population Based National Tuberculosis Prevalence Survey among Adults (>15 Years) in Pakistan, 2010-2011. *PLoS One* 2016; **11**(2): e0148293.
